# Population-level integration of single-cell datasets enables multi-scale analysis across samples

**DOI:** 10.1038/s41592-023-02035-2

**Published:** 2023-10-09

**Authors:** Carlo De Donno, Soroor Hediyeh-Zadeh, Amir Ali Moinfar, Marco Wagenstetter, Luke Zappia, Mohammad Lotfollahi, Fabian J. Theis

**Affiliations:** 1 Institute of Computational Biology, Helmholtz Center Munich, Munich, Germany; 2 School of Life Sciences Weihenstephan, Technical University of Munich, Munich, Germany; 3 School of Computing, Information and Technology, Technical University of Munich, Munich, Germany; 4 Wellcome Sanger Institute, Wellcome Genome Campus, Cambridge, UK

**Keywords:** Data integration, Machine learning, Software, Transcriptomics

## Abstract

The increasing generation of population-level single-cell atlases has the potential to link sample metadata with cellular data. Constructing such references requires integration of heterogeneous cohorts with varying metadata. Here we present single-cell population level integration (scPoli), an open-world learner that incorporates generative models to learn sample and cell representations for data integration, label transfer and reference mapping. We applied scPoli on population-level atlases of lung and peripheral blood mononuclear cells, the latter consisting of 7.8 million cells across 2,375 samples. We demonstrate that scPoli can explain sample-level biological and technical variations using sample embeddings revealing genes associated with batch effects and biological effects. scPoli is further applicable to single-cell sequencing assay for transposase-accessible chromatin and cross-species datasets, offering insights into chromatin accessibility and comparative genomics. We envision scPoli becoming an important tool for population-level single-cell data integration facilitating atlas use but also interpretation by means of multi-scale analyses.

## Abstract (web summary)

By learning representations for both cells and various condition covariates, scPoli facilitates atlas-level integration and analysis of single-cell genomics datasets with improved interpretability.

## Main

The advancements in single-cell technologies have enabled the generation of datasets comprising information from millions of cells. These datasets, also called ‘atlases’, include data from different conditions and individuals and offer precious insight into cellular processes and states in different scenarios. Consortia such as the Human Cell Atlas^1^ and the Human BioMolecular Atlas Program^2^ aim to generate organ- and body-level atlases that allow one to study human organs from development to aging in healthy and disease samples. A possibility opened by these atlases is that of meta-analyses relating cell types and states with biological conditions or demographics metadata^3,4^.

Performing meta-analysis on an atlas requires learning a joint representation of all datasets correcting batch effects between them^5–7^. Tremendous efforts have been made to solve the data integration problem for single-cell RNA sequencing datasets using approaches ranging from statistical^8–11^ and graph-based^12–14^ methods to deep learning models^5,15–17^. Nonetheless, single-cell data integration remains challenging^18^, especially in the case of many datasets with a variety of technical and biological properties.

Many analyses can be accelerated by mapping data on top of an atlas. Algorithms for efficient use of reference atlases are known as reference mapping methods^19–21^, which build upon data integration algorithms to update an existing atlas by integrating a query dataset. Transferring information from reference to query enables efficient annotation of the query cells^4,20,22,23^.

Existing deep learning integration methods^6^ rely on one-hot-encoded (OHE) vectors to represent conditions^15,24^. This encoding does not allow a downstream interpretation of the effect of each sample on the mapping. Additionally, in the presence of many unique condition categories, the number of conditional inputs can become close or equal to the number of gene expression measurements leading the model to produce inaccurate data representation^25^. Among current reference-building methods^10,12,24,26^ only scANVI and Seurat v3 offer cell type classification coupled with a reference mapping algorithm^19,21^. Yet, while they can integrate annotated data to extend the reference, this requires retraining, which is time-consuming and can sometimes be not possible due to data sharing restrictions.

In this Article, we introduce single-cell population level integration (scPoli), a semi-supervised conditional generative model and open-world learner^5,27^ combined with advances in meta-learning^28^ that is able to learn representations for both cells and samples (or other batch covariates). scPoli offers a cell-level and a sample-level view of the dataset enabling multi-scale analysis: the simultaneous exploration of sample and cell representations.

scPoli uses prototype-based cell label transfer and is augmented with an uncertainty estimation mechanism. We demonstrate that scPoli is competitive in data integration and cell annotation with other methods across six datasets. We further showcase the features of our model by integrating a lung atlas and performing reference mapping for two queries. We show potential use cases of condition embeddings such as sample classification and data integration workflow guidance. Finally, we build a reference of 7.8 million peripheral blood mononuclear cells (PBMCs) from 2,375 samples and explore the sample-level representation obtained with scPoli.

## Results

### scPoli learns joint cell and sample representations

**Fig. 1:**
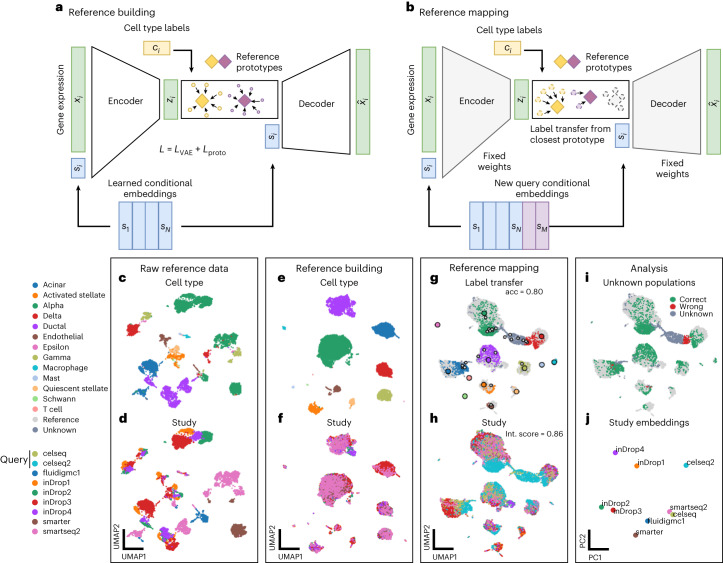
scPoli enables learning cell-level and sample-level representations. **a**, scPoli reference building: the model integrates different datasets and learns condition embeddings for each integrated study and a set of cell type prototypes. **b**, scPoli reference mapping: the model weights are frozen (in gray) and a new set of condition embeddings are added to the model. Cell type labels are transferred from the closest prototype in the latent space. Example of a standard workflow using scPoli on multiple pancreas datasets. **c**,**d**, Uniform manifold approximation and projection (UMAP) of the raw data to be integrated in a reference (13,093 cells), showing cell types (**c**) and studies (**d**) by color. **e**,**f**, Integrated reference data colored by cell type (**e**) and study (**f**). **g**, A total of 3,289 query cells (celseq and celseq2 studies) are projected onto the reference data in the reference mapping step. UMAPs show in color the query cells and in gray the reference cells. Reference cell type prototypes are shown in bigger circles with a black edge. Unlabeled prototypes are shown in bigger gray circles with black edges. The accuracy of the label transfer is 80%. **h**, Cells are colored by study or origin after reference mapping. The model achieves a mean integration score of 0.86. **i**, Outcome of the label transfer step from reference to query. **j**, PCA of the condition embeddings learned by scPoli.

The variation of gene expression (**x**_*i*_) in a dataset can be ascribed to batch effects and biological signals. Similarly to other conditional models^15,29^, scPoli aims to regress out batch effects in a nonlinear fashion by means of a conditional variable (**s**_*i*_) representing batch while retaining biological information. Moreover, scPoli posits that cell identities (*k*_*i*_) can be represented with learnable cell type prototypes^28^ modeled using latent cell representations (**z**_*i*_) (Fig. 1a). scPoli, therefore, introduces two modifications to the conditional variational autoencoder (CVAE) architecture widely used for data integration^5,15,24^ and perturbation modeling^16,29^ in single-cell genomics. These modifications are (1) the replacement of OHE vectors with continuous vectors of fixed dimensionality to represent the conditional term, and (2) the usage of cell type prototypes to enable label transfer.

CVAE-based methods encode each condition by means of OHE representations ${{\bf{c}}}_{1:N}^{{{\mathrm{OHE}}}}\in {{\mathbb{R}}}^{N}$, where *N* is the number of conditions. These are concatenated to the input, and an additional neuron for each condition is added to the first layer of the encoder neural network. In the case of thousands of conditions to be integrated, this approach leads to an increase in the number of total trainable parameters that can slow down training. scPoli uses learnable condition embeddings ${{\bf{s}}}_{1:N}^{{{\mathrm{emb}}}}\in {{\mathbb{R}}}^{E}$ of fixed dimensionality. These are concatenated to the input and learned at training time (Fig. 1a). As a result, scPoli is more scalable than a CVAE in scenarios where many conditions are to be integrated (see ‘Scalability analysis’ in Methods). Furthermore, these condition embeddings capture meaningful representations of each condition and can be analyzed, providing insight in large-scale studies. scPoli can also be used to integrate multiple conditions simultaneously. This is achieved by modeling condition covariates using independent embeddings. scPoli can perform reference mapping by freezing the weights of the model trained on the reference and learning a new set of *M* embeddings to accommodate the query data conditions (Fig. 1b).

The second addition to CVAE models is the incorporation of prototypes used in meta-learning^30^. These allow efficient learning across tasks and datasets and have been used for cell type classification^26^. scPoli models prototypes using the average latent representation of cells belonging to the same cell type and leverages them to transfer annotations and improve data integration by means of an additional term in the learning objective called prototype loss. This term encourages the model to reduce the distance between the latent representation of a cell and its prototype (Methods). We show this leads to better preservation of biological signals. Unlabeled cells are classified by comparing distances to the prototypes, and the label of the closest prototype is assigned as a predicted cell type label. We also exploit the distance of each cell to its closest prototype as a proxy for uncertainty. Finally, prototypes enable extending an initial reference atlas with novel cell types from a labeled query without retraining the reference model as opposed to existing methods^24^.

We illustrate a standard scPoli workflow on a collection of nine pancreas studies (see ‘Benchmark datasets’ in Methods) in Fig. 1c–j. We build an integrated reference on seven datasets. We use two datasets (celseq and celseq2) as an unlabeled query and map them onto the reference data. We can observe that query cells are mapped onto the reference data (mean integration score of 0.86) and that most cells are classified correctly with an accuracy of 80%. A cluster of cells (beta cells) that was removed from the reference dataset to mimic an unknown cell type scenario is correctly identified. After a principal component analysis (PCA) we can observe that the condition embeddings learned by scPoli capture similarities between the integrated samples.

### scPoli accurately integrates datasets and transfers annotations

To understand how well scPoli integrates single-cell datasets, and how accurately it transfers cell type annotations, we benchmarked our model against other methods for data integration and label transfer. We included in this comparison both deep learning models (scVI^15^, scANVI^24^ and MARS^26^) and other types of methods (Seurat v3^12^, Symphony^20^ and a linear support vector machine (SVM)). Out of these models, only our method, scANVI and Seurat v3 tackle both data integration and label transfer, while some exclusively do integration (scVI and Symphony), or classification (MARS and SVM). All of these models, except for MARS and Symphony were part of the Luecken et al.^6^ data integration benchmark, where they came out as top performers.

We tested these methods on six datasets, spanning a variety of scenarios (see ‘Benchmark datasets’ in Methods) (Supplementary Fig. 1). For each dataset a set of studies to use as reference was picked, while the rest was used as query. To quantify the performance of data integration we used metrics to assess biological conservation and batch correction proposed in ref. ^6^ (Methods).

**Fig. 2:**
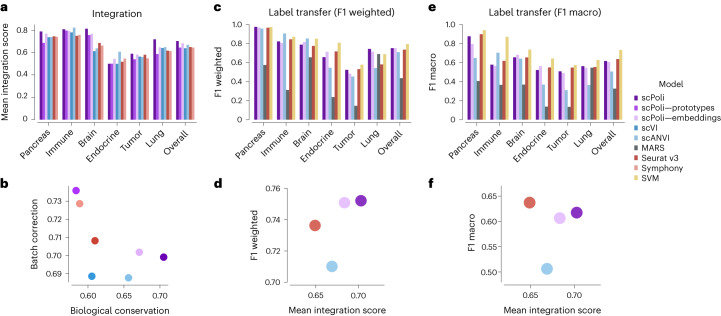
scPoli reaches state-of-the-art performance on data integration and label transfer. **a**, Mean integration score obtained using the benchmarked models on different datasets. The bars on the right show the average results across datasets. **b**, Overall scores across datasets for biological conservation and batch correction performance of the benchmarked models. **c**, Weighted F1 scores achieved by each model when classifying query cells on the various datasets. **d**, Weighted F1 score and the overall integration score of the models capable of both data integration and label transfer. **e**, Macro-averaged F1 query classification scores achieved by each model on the various datasets. **f**, Macro-averaged F1 score and the overall integration score of the models capable of both data integration and label transfer.

We found that scPoli outperformed the next best-performing model (scANVI) by 5.06% in data integration (Fig. 2a). When we looked separately at batch correction and biological conservation metrics, we observed that scPoli preserved biologically meaningful signals better than other methods. To understand whether the improvements stemmed from the use of condition embeddings or from the inclusion of the prototype loss, we benchmarked two variants of our model. We included a scPoli model with standard OHE vectors to represent batch, and a scPoli model trained without prototype loss. We found the prototype loss to be the driver of the improvement in biological conservation (Fig. 2b).

To assess the quality of the classification achieved during reference mapping we used two metrics: the weighted averaged and the macro-averaged F1 score, with the latter being more sensitive to underrepresented cell types. We observed that scPoli outperformed all methods except for the linear SVM on the weighted F1 metric (Fig. 2c). Out of the models that are capable of both data integration and cell type classification, scPoli came out on top (Fig. 2d). When looking at the macro-averaged F1 score, scPoli showed comparable performance to Seurat v3 and a sizeable improvement over scANVI, indicating better performance on underrepresented cell types (Fig. 2e,f). The SVM was the best-performing method according to this metric, a result corroborated by previous work in the field^31^.

Furthermore, scPoli’s integration performance and label transfer accuracy were stable across runs and different dataset sizes (Methods and Supplementary Fig. 2).

### scPoli enables interpretable atlas-level integration

**Fig. 3:**
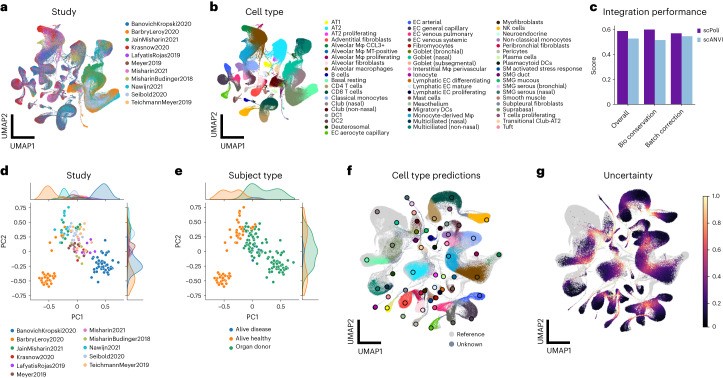
scPoli performs interpretable integration and query-to-reference mapping on the HLCA. **a**,**b**, Uniform manifold approximation and projection (UMAP) of the integrated HLCA core after reference building, cells are color coded by their study of origin (**a**) and by cell type (**b**). DC, dendritic cell. EC, endothelial cell. NK, natural killer cell. **c**, Comparison of integration performance yielded by scPoli and the scANVI. **d**,**e**, Visualization of the first two PCs obtained with a PCA of the sample embeddings learned from the reference data. Samples are color coded by their original study (**d**) and by sample type (**e**). **f**, UMAP of the joint query and reference datasets after query-to-reference mapping for a healthy query. Reference cells are shown in light gray, query cells are colored by the predicted cell type and unknown cells are shown in dark gray. Legend for the predicted cells is shared with **b**. Reference prototypes are shown as bigger dots with a black border, and are colored by cell type. **g**, UMAP of the integrated object with uncertainties in color. Reference cells are shown in gray. Labeled cell type prototypes are shown with bigger dots with a black border.

We showcase the data integration capability and quality of label transfer yielded by scPoli on the Human Lung Cell Atlas (HLCA)^4^, a curated collection of 46 datasets of the human lung, with samples from 444 individuals. The atlas is divided into a core collection of data, which comprises data from 166 samples and 11 datasets, and an extended one that includes the remaining data. Following the work in the original study, we used the HLCA core data for reference building. We integrated data at the sample level to obtain a better resolution of the condition embeddings and allow interpretation using sample metadata. For prototype training we used the finest annotations, resulting in 58 prototypes. We observed that scPoli successfully integrated data from different studies (Fig. 3a) while maintaining structure among the known cell identities (Fig. 3b). We further assessed the quality of data integration and compared it against scANVI. To keep the comparison consistent, we also trained scANVI at the sample level. scPoli yielded an integration that preserved biologically meaningful variation better than scANVI, while achieving similar performance in batch correction (Fig. 3c).

When looking at the first two principal components (PCs) of the sample embeddings we found that samples from the same studies grouped together (Fig. 3d and Supplementary Fig. 3a). We additionally found metadata that covaried with the sample representation. These included information regarding properties of the sample such as subject type (for example, donor or alive) (Fig. 3d) and anatomical location (Supplementary Fig. 3b). Other covariates (for example, sex or ethnicity) appeared to be mixed in this space (Supplementary Fig. 3c,d), indicating that the main drivers behind batch effects are likely to be related to the nature of the tissue, its processing and other technical factors.

### scPoli propagates high-resolution annotations

One of the main hurdles in atlas building comes from discrepancies in annotation terms across datasets. scPoli and other methods that leverage these annotations require prior label harmonization labels before usage, which requires expert knowledge. Nonetheless, scPoli can work on multiple levels of annotation (for example, from coarse to fine) and can propagate labels to underclustered datasets during reference building. scPoli is able to model multiple sets of prototypes for each level of annotations. This prior information is then leveraged by optimizing the prototype loss on each set of labeled prototypes. To simulate such a scenario, we integrated the HLCA, but this time using both a coarse and a fine annotation (Supplementary Fig. 4a–c). Additionally, for one dataset in the reference (Krasnow2020), we kept only the coarse annotation. We then used scPoli to propagate high-resolution labels to these cells obtaining an overall accuracy of 84.4% (Supplementary Fig. 4d).

### scPoli enables query-to-reference mapping and label transfer

After building a reference using the HLCA core dataset, we mapped a group of healthy samples (Meyer, 2021)^32^ (Fig. 3f). These data consist of six samples and contain nine cell identities not present in the reference. As a proxy for uncertainty in cell type prediction, we use the Euclidean distance from the closest prototype in the reference. Similarly to the original HLCA study, in which a *k*-nearest neighbor (kNN) graph-based uncertainty was used, we noticed that cells that lay in regions of transitions between cell types displayed the highest levels of uncertainty, as well as cells whose identities were not present in the reference data (Fig. 3g). We considered all cells with an uncertainty higher than the 90% quantile as unknown (Supplementary Fig. 5a) and inspected the classification performance by cell type (Supplementary Fig. 5b). A subset of novel cells were successfully detected as unknown, especially chondrocytes, erythrocites and myelinating and non-myelinating Schwann cells. Natural killer T cells, GammaDelta T cells and regulatory T cells were not detected as unknown and were mostly classified as either CD4 T cells or CD8 T cells, which could also be a result of overclustering in the original atlas^33^. Overall, scPoli achieved an accuracy of 75%, outperforming the model used in the original study, which yielded an accuracy of 69%. Label transfer in scPoli happens without the need for the reference dataset. scPoli transfers labels by comparing distances to a small set of prototypes that are obtained during the reference building step and stored within the reference model. This constitutes a big advantage in cases where the reference data cannot be shared. Furthermore, we observed that scPoli is more robust at detecting unknown cells than the methodology involving a kNN graph and scANVI. We compared the ratio of true predictions across different thresholds for unknown cell type detection for three models and scPoli consistently obtained better accuracy (Supplementary Fig. 5c).

To see how scPoli would perform when mapping a query dataset from a different condition than the one in reference, we mapped data collected from cancer patients. These data contain two cell identities not present in the reference (cancer and erythrocytes). We observed that scPoli successfully mapped the query dataset (Supplementary Fig. 6a). Since this query has a much coarser cell type annotation, we mapped the labels obtained with scPoli to the cell types present in the query via a mapping obtained from the authors of the study. We observed that almost all cancer cells mapped to a cluster whose label prediction had high uncertainty and was classified as unknown (Supplementary Fig. 6b,c). We observed that 85% of cancer cells and 98% of erythrocytes were identified as unknown (Supplementary Fig. 6d). Also in this case, scPoli obtained better accuracy across different thresholds for unknown cell type detection compared to a kNN classifier and scANVI (Supplementary Fig. 6e).

### scPoli enables multi-scale classification of cells and samples

**Fig. 4:**
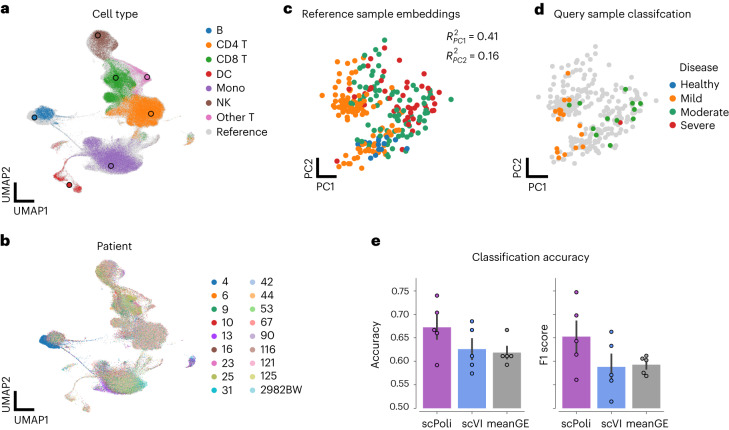
scPoli allows classification of disease state for unlabeled samples. **a**, Uniform manifold approximation and projection (UMAP) of Su et al. dataset after integration. Unlabeled cells are shown colored by the predicted cell type. Labeled cell type prototypes are shown with bigger dots with a black border. DC, dendritic cell. NK, natural killer cell. **b**, UMAP of the integrated dataset colored by patient. Data from 30 random samples out of 270 are shown to simplify the visualization. **c**, PCA of the labeled sample embeddings obtained with scPoli colored by disease state. **d**, Unlabeled sample embeddings are shown in PCA space colored by their predicted disease state on top of the reference sample embeddings in gray. **e**, Comparison of classification accuracy and F1 score obtained on scPoli embeddings and average gene and scVI latent expression vectors for each sample (pseudobulks). Data points are obtained from cross-validation (*n* = 5). Data are presented as mean values ± standard error of the mean.

We tested unlabeled sample classification as a potential use case for condition embeddings. We integrated a coronavirus disease 2019 (COVID-19) PBMC dataset by Su et al.^34^ with large biological signals using the sample covariate as condition. The data contains 559,517 cells from 270 samples across various states of COVID-19. We selected 30 random samples and set their cell type annotations and sample annotations as unknown. We then integrated the data and propagated the labels. We first assessed the quality of integration and label transfer, which achieved an accuracy of 90% (Fig. 4a,b).

The sample-level metadata are organized into four classes: healthy, mild, moderate and severe. The sample embeddings of the reference data showed variation associated with this phenotypic covariate in the two first PCs (adjR^2^_PC1_ 0.41, adjR^2^_PC2_ 0.16 obtained with an ordinal regression; the adjusted *R*^2^ is reported to take into account the different numbers of available predictors) (Fig. 4c). We, therefore, proceeded to classify the disease state of the query samples using a kNN classifier trained on the reference sample embeddings. This classification yielded an accuracy of 73%. We compared the performance and stability of this sample classification against that obtained with a classifier trained on sample pseudobulks, and one trained on vectors of average latent expression per sample after integration with scVI. We did so by splitting the training data into labeled and unlabeled in a five-fold cross-validation setting. When we compared the accuracy and F1 score obtained by a classifier trained on scPoli sample embeddings we observed that these were better than those obtained using the other classifiers, with weighted F1 scores of 65.3 ± 2.5%, 59.3 ± 1.3% and 58.8 ± 2.1%, for scPoli sample embeddings, mean gene expression and mean scVI latent expression, respectively (Fig. 4e).

### scPoli supports experimental design in integration workflows

**Fig. 5:**
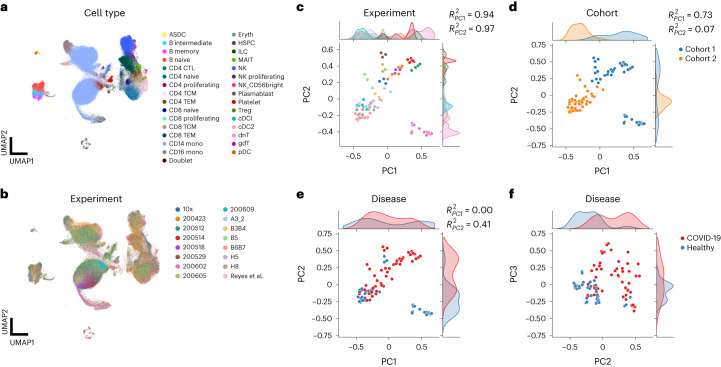
scPoli sample embeddings capture technical variation and can guide data integration workflows. **a**,**b**, Uniform manifold approximation and projections (UMAPs) of Schulte-Schrepping et al. dataset consisting of healthy and COVID-19 PBMC samples after sample-level integration. Cells are colored by cell type (**a**) and experiment (**b**), respectively. DC, dendritic cell. NK, natural killer cell. HSPC, hematopoietic stem and progenitor cell. CTL, cytotoxic T cell. TCM, central memory T cell. TEM, effector memory T cell. ILC, innate lymphoid cell. MAIT, mucosal-associated invariant T cell. **c**–**f**, Sample embeddings obtained with scPoli colored by experiment (legend shared with **b**) (**c**), cohort (**d**) and disease (**e** on principal components 1 and 2 and **f** on principal components 2 and 3). For each covariate the association with the first and second PCs is displayed.

To understand the relationship between technical and phenotypic factors in scPoli’s sample embedding we integrated another COVID-19 dataset consisting of 222,003 cells (Schulte-Schrepping et al.)^35^, with 99 samples from 65 patients in two different cohorts (Fig. 5a,b) obtained from a multitude of experiments with different technical properties. When we examined the sample embedding, we observed the major sources of variation to be related to technical factors, suggesting that these play a bigger role in batch effects. Indeed, the first two PCs of the sample embedding were explained by the experiment (adjR^2^_PC1_ 0.94, adjR^2^_PC2_ 0.97) (Fig. 5c) and the cohort from which the samples were obtained (adjR^2^_PC1_ 0.73) (Fig. 5d), rather than the disease state (adjR^2^_PC1_ 0.00) (Fig. 5e). We could find association with disease state, but only in further PCs (adjR^2^_PC2_ 0.41, Fig. 5e,f).

These analyses suggest that, while in more focused studies where technical factors are controlled and kept consistent across samples, biological signals represent the main source of sample-level heterogeneity; in bigger-scale collections of data with a variety of technical factors, these variations will dominate. This led us to speculate that, since sample embeddings identify major sources of variations in the data, they could also guide the choice of the conditions to integrate in a data integration workflow. We proceeded to integrate the data using other two models conditioned on the covariates that showed association with the first PC of the embedding space (experiment and cohort).

Additionally, we trained a model with a dummy batch covariate, which was equal for all cells. In this case, scPoli will leverage exclusively cell type annotations and the prototype loss to perform integration. We observed that the integration yielded by the cohort- and experiment-level models displayed a similar quality of integration despite the reduction in the number of conditions to integrate (2 for the cohort covariate, 16 for experiment, compared to 99 for sample) (Supplementary Fig. 7). All models conditioned on an actual batch variable outperformed the one trained on a dummy covariate. This demonstrates an important use case of this sample-level representation. Revealing the main sources of undesired batch effects can in fact lead to faster data integration workflows and potentially improve the quality of the integrated cell representations by selecting the most appropriate batch covariate.

### scPoli can model multiple batch covariates

scPoli can model multiple batch covariate using independent embeddings which are then concatenated to the gene expression input. Doing so will yield a cell embedding and an embedding space for each batch condition. We applied this workflow on the Schulte-Schrepping dataset, where we integrated the data using both ‘experiment’ and ‘sample’ covariates (Supplementary Fig. 8a,b). This new model yielded experiment-level embeddings that varied according to cohort and disease information in the two first PCs (Supplementary Fig. 8c–e). In addition to this, scPoli produced sample embeddings where the experiment-level variation was mitigated in comparison to the one observed in the model conditioned only on samples, suggesting that scPoli disentangled experiment-level variation from sample-level variation (Supplementary Fig. 8f–h).

### scPoli can integrate data and transfer labels across species

Integrating data collected from different species can be a challenging task for data integration models. To understand how scPoli would perform in such a scenario, we built a reference from cells collected from the primal frontal cortex of marmoset and mouse using ortholog genes^36^. scPoli was able to integrate the data from the two species and map cells of the same cell type together (Supplementary Fig. 9a,b). We then performed reference mapping and label transfer using cells from human data as a query. scPoli was able to perform query-to-reference mapping across different species and yielded an overall label transfer accuracy of 86% (Supplementary Fig. 9c–e).

### scPoli can be extended to further data modalities

In this work, we focus on the applications of scPoli on scRNA-seq data; nonetheless, with the appropriate adaptations, scPoli can be applied to other modalities. To demonstrate this, we used scPoli to integrate a set of single-cell sequencing assay for transposase-accessible chromatin (scATAC-seq) samples by modeling the likelihood of the input data using a Poisson distribution, as proposed in ref. ^37^. We tested this on the NeurIPS 2021 multimodal data integration dataset^38^, from which we used the scATAC-seq modality. scPoli integrated data from different samples (Supplementary Fig. 10a) and yielded condition embeddings that captured similarities between samples generated at the same site (Supplementary Fig. 10b). We assessed the quality of this integration by comparing it against that of PeakVI^39^, a CVAE-based method for scATAC-seq data integration, and found that scPoli achieved comparable performance (Supplementary Fig. 10c).

### scPoli scales to datasets with thousands of samples

**Fig. 6:**
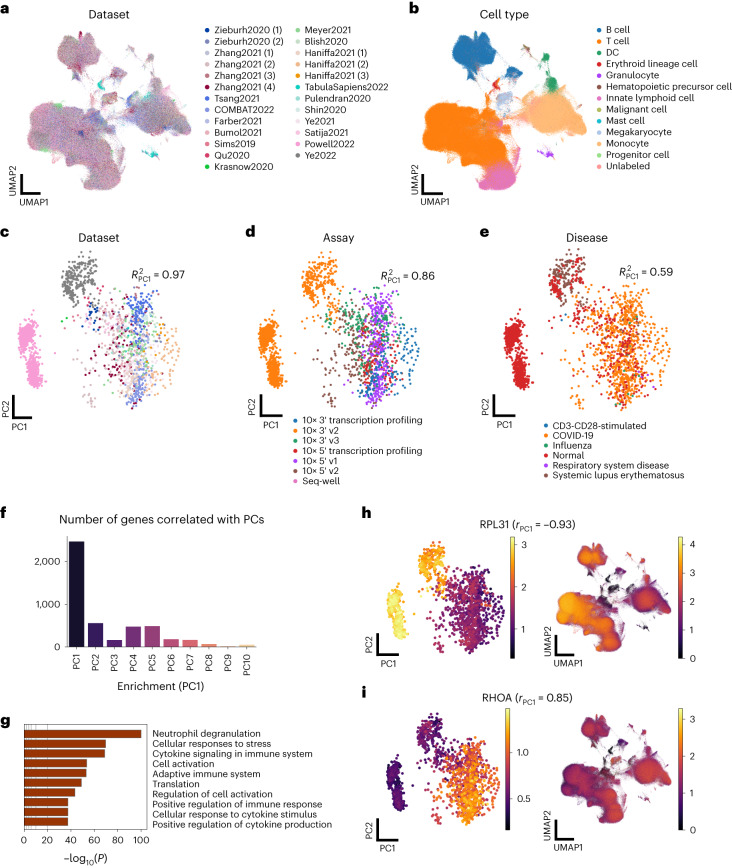
scPoli enables population-level integration of 7.8 million cells from 2,375 PBMC samples. **a**, Uniform manifold approximation and projection (UMAP) of the PBMC atlas after integration (subset to 1 M cells). Colors show different datasets of origin. **b**, The same UMAP colored by cell type. **c**–**e**, Sample embeddings projected onto the two first PCs and colored by dataset of origin (legend shared with **a**) (**c**), assay (**d**) and disease (**e**). The displayed *R*^2^ is the adjusted *R*^2^ obtained by fitting a linear model on the first PCs and the covariates. **f**, Number of genes significantly (*P* < 0.01 and |*r*| > 0.3) correlated with the PCs of the sample embedding space. **g**, Biological process and pathway enrichment analysis of the genes found to be significantly correlated with PC1. **h**,**i**, RPL31 (**h**) and RHOA (**i**) expression patterns in the sample embedding space (left) and cell embedding space (right). These genes were respectively among the most negatively and positively correlated ones with PC1.

We further leveraged scPoli to build a PBMC atlas comprising roughly 7.8 million cells from 2,375 samples, 1,977 subjects and 25 datasets. We obtained the integrated cell representation (Fig. 6a,b and Supplementary Fig. 11a) and the sample embeddings, which we analyzed to examine the dominant sources of variations across samples. We observed that, while most of the variance was explained by the first PC of this space, substantial signal was still present in the further PCs, suggesting that scPoli makes full use of this space to encode information useful for batch correction (Supplementary Fig. 11b). We found the first PC to be mainly associated with the dataset of origin of the sample (Fig. 6c and Supplementary Fig. 11c,d). We used a linear model to quantify this association, which yielded an adjusted *R*^2^ of 0.97. We also observed a strong association with sequencing assay (adjR^2^ 0.86) (Fig. 6d and Supplementary Fig. 11g,h) and moderate association with disease phenotype (adjR^2^ 0.59) (Fig. 6e and Supplementary Fig. 11e,f). When we looked at how information such as sex and ethnicity mapped onto the sample embedding obtained with scPoli, we observed no clear patterns in the embedding space (Supplementary Fig. 11i–n). We compared the structure in scPoli sample embeddings with that obtained on vectors of average gene expression by sample, and observed that, while some of the patterns showed similarities, scPoli was more sensitive to differences between datasets and preserved more structure overall (Supplementary Fig. 12).

To understand whether the sample embeddings reflected any gene expression patterns across samples, we computed the Pearson correlation between the mean expression of each gene in the various samples and the PC scores of the embeddings. We thus obtained lists of significantly correlated genes with each PC (*P* < 0.01) and filtered them for coefficients of determination larger than 0.3 in absolute value. We found the biggest number of correlated genes with the first PC. This number did not decrease regularly going further through the PCs, and we found PC2, PC4 and PC5 to also have a substantial number of correlated genes (Fig. 6f). When we looked at which genes were most strongly correlated with PC1, we observed a strong presence of ribosomal genes: 14 out of the 15 top negatively correlated genes. This was reflected also in a general association of PC1 with the mean ribosomal gene fraction of each sample (Supplementary Fig. 13a). On the other hand, we did not observe a clear association with mitochondrial gene fraction (Supplementary Fig. 13b). We performed a biological process and pathway enrichment analyses of the genes correlated with PC1 and found terms related to the immune and stress response, cytokine signaling, neutrophile degranulation and cell activation (Fig. 6g). The top negatively correlated gene with PC1 was *RPL31* (*r* = −0.93), which is a ribosomal gene involved in the cellular response to stress; the second top positively correlated gene, on the other hand, was *RHOA* (*r* = 0.85), a gene involved in the immune response and that we observed to be more highly expressed in disease samples (Supplementary Fig. 13c). These findings reflect the associations found with both technical and phenotypic covariates and PC1. When we looked at the patterns of expression of these genes in the sample and cell embedding spaces, we observed that scPoli successfully mixed these signals in the integrated cell representation but also offered the unique feature of exploring them in the sample embedding space where they were preserved (Fig. 6h,i). A similar enrichment analysis with PC2- and PC4-correlated genes revealed terms related to RNA and DNA metabolism in the first case (Supplementary Fig. 13d), and response to stress and cytokine production in the second (Supplementary Fig. 13f). We did not perform this analysis with PC3, due to the low number of correlated genes. We show the expression patterns in both cell and sample embedding spaces of genes associated with PC2 (*SSR2*) and PC4 (*TSLPY2*) in Supplementary Fig. 13f,g.

We believe that the multi-scale representation obtained by scPoli could represent a useful tool for researchers to understand which genes drive batch effects the most or are affected by technical factors in the data generation process.

## Discussion

We have presented scPoli, a generative model for data integration, label transfer and reference mapping. scPoli learns representations of the input data at different scales by learning cell and sample embeddings. This enables multi-scale analyses whereby the user can explore sample information in a dedicated latent space, while still having access to an integrated single-cell object. By freezing the weights of the model and learning new embeddings, scPoli is able to quickly map newly generated data onto a previously built reference.

We have shown that scPoli is competitive with state-of-the-art methods for data integration and label transfer. Thanks to the use of cell type prototypes, scPoli consistently preserved biological information better than other methods. Moreover, scPoli performs label transfer in a privacy-aware fashion without the need for the reference data. We illustrated the features of our model by integrating the HLCA. scPoli outperformed the model used in the original study in data integration and yielded a sample latent representation that reflected similarities between different samples. We also showcased our model’s reference mapping capabilities by mapping an unlabeled query dataset of healthy samples and one of cancer samples. Furthermore, we demonstrated the viability of scPoli as a scATAC-seq data integration method and its ability to perform integration and reference mapping across species.

To understand better the information captured by the sample embeddings and potential use cases we investigated three further datasets. Our findings suggest that in smaller-scale studies scPoli reveals phenotypical sources of variation and can enable multi-scale classification of both cell types and samples. Nonetheless, as the complexity and number of samples increase, the sample embeddings obtained with our model are more likely to reflect variations of technical nature. In these cases, scPoli’s sample embeddings can help identify the main sources of technical variation driving the batch effects. This can be used to guide data integration workflows by identifying the most appropriate covariates to use as batch condition and to discover gene expression patterns across samples associated with batch effects and technical and phenotypic factors. Furthermore, scPoli can integrate and model multiple batch covariates, which will yield multiple batch embedding spaces and can improve interpretability by disentangling dataset-level variation from sample-level variation.

scPoli, like other methods for data integration that leverage CVAE, provides the user with a lower-dimensional single-cell integrated object and not a corrected count matrix. Moreover, the quality of the integration will be a function of the number of samples that can be used in the reference-building step. As the model integrates samples with different technical or phenotypical characteristics, it does a better job at regressing out the batch effects.

A limitation of models that make use of cell type information is the need for high-quality and harmonized annotation across datasets. scPoli is also susceptible to this and requires cell type label harmonization before reference building.

We found that the use of prototypes improves the preservation of biological information. We use distances from these prototypes and a latent cell embedding to transfer labels and yield an uncertainty associated with it. While latent representations obtained with VAEs are learned on smoother manifolds than vanilla autoencoders, this linear approximation remains suboptimal. Nonetheless, this approach has been used in foundational work in generative modeling research^40–42^. This limitation becomes relevant for our uncertainty, whose distribution can vary substantially in different scenarios. Therefore, we recommend users visualize these distributions and choose the best threshold for detecting unknown cells manually.

We recommend care when interpreting the sample-level representations obtained with scPoli. The main sources of variation between samples will change across datasets. Different covariates are likely to explain these variations in different datasets. This will determine which are the most sensible use cases for sample embeddings.

We believe scPoli will be useful as a tool for data integration and reference mapping given its improvements in the conservation of biological signals. Furthermore, we expect scPoli’s sample-level embeddings to provide researchers with another point of view over large-scale datasets, and pave the way to multi-scale analyses that investigate and link patterns at different scales. Single-cell atlassing is entering the stage of population-level studies, which implies the need for models across this level of variation.

## Methods

### scPoli

scPoli is a semi-supervised generative deep learning method comprising two components, an unsupervised backbone based on CVAEs^27^ and a cell type supervised component leveraging prototype networks^28^. In the following we first outline the data generation process describing different inputs for the model. Following that, we will discuss details about the main components of scPoli and the training procedure.

### Notation

We denote a collection of single-cell data from different samples $\{{X}_{1},\ldots ,{X}_{n}\}$ with sample labels $\{{c}_{1},\ldots ,{c}_{n}\}$. Within each sample we have $X=\{{{\bf{x}}}_{1},\ldots ,{{\bf{x}}}_{j}\}$ cells with cell type annotations $\{{a}_{1},\ldots ,{a}_{j}\}$ and sample annotation *c*. A batch of data passed to the model during training will contain the single-cell gene expression of a random sample of cells from the training data, plus the sample and cell type labels associated with each cell $\{{{\bf{x}}}_{i},{c}_{i},{a}_{i}\}$. When we pass multiple condition covariates and cell type annotations to the model this becomes $\{{{\bf{x}}}_{i},{{\bf{c}}}_{i},{{\bf{a}}}_{i}\}$.

### CVAEs

Variational autoencoders (VAEs)^40^ are generative models that employ variational inference and deep neural networks to learn the underlying distribution of the data they are trained on. These models consist of an encoder network that parameterizes the latent variational distribution of the data and a decoder network that samples from this distribution and maps the data back to the input space. CVAEs^15^ are an extension of such models in which the input data is conditioned on another random variable. CVAEs aim to maximize the likelihood of the data, formulated following the Bayes chain rule as

1 $${p}_{\theta }({\mathbf{x}}|{\mathbf{c}})={\int }_{\mathbf{z}}{p}_{\theta }({\mathbf{x}}|{\mathbf{z}},{\mathbf{c}}){p}_{\theta }({\mathbf{z}}|{\mathbf{c}}){\mathrm{d}}{\mathbf{z}},$$

where **x** is the input gene expression data, **z** is the latent variable that is assumed to parameterize the latent distribution of the data, **c** is the condition variable, and *θ* is the parameters of the model. Since the likelihood as formulated above is in most cases intractable, VAEs use amortized inference by approximating the posterior distribution by means of a neural network ${p}_{\theta }\left({\bf{z}}{\rm{|}}{\bf{x}},{\bf{c}}\right)\sim {q}_{\phi }\left({\bf{z}}{\rm{|}}{\bf{x}},{\bf{c}}\right)$, where *ϕ* are the parameters of said network. The loss used to optimize these models aims to jointly reduce the reconstruction error between input and output and make ${q}_{\phi }\left({\bf{z}}{\rm{|}}{\bf{x}},{\bf{c}}\right)$ as close as possible to ${p}_{\theta }\left({\bf{z}}{\rm{|}}{\bf{x}},{\bf{c}}\right)$.

The resulting loss function, also known as evidence lower bound, is formulated as

2 $${\mathcal{L}}_{\mathrm{CVAE}}(\theta ,\phi )=-{\mathbb{E}}_{{\bf{z}}\sim {q}_{\phi }({\bf{z}}|{\bf{x}},{\bf{c}})}\log {p}_{\theta }({\bf{x}}|{\bf{z}},{\bf{c}})+{D}_{\mathrm{KL}}({q}_{\phi }({\bf{z}}|{\bf{x}},{\bf{c}})||p({\bf{z}}))\left)\right.\!,$$

where *θ* and *ϕ* are respectively the encoder and decoder networks parameters, ${\mathbb{E}}$ is the expectation and *D*_KL_ indicates the Kullback–Leibler divergence.

The first term of the loss formulated above is also known as reconstruction loss, and it might take different forms depending on the generative process of the input data. In the case of count data, we assume a negative binomial distribution as input; thus the likelihood will follow:

3 $$\begin{array}{cc}{p}_{\theta }\left({\bf{x}}{\rm{|}}{\bf{c}}\right) & =\text{NB}\left({\boldsymbol{\mu }},{\boldsymbol{\alpha }}\right).\end{array}$$

In the case of scATAC-seq data we assume a Poisson distribution:

4 $$\begin{array}{cc}{p}_{\theta }\left({\bf{x}}{\rm{|}}{\bf{c}}\right) & =\text{Poisson}\left({\boldsymbol{\lambda }}\right).\end{array}$$

The negative log-likelihood of the appropriate distribution is used as reconstruction loss during training.

### Condition embeddings

The architectural backbone of scPoli builds upon CVAEs, but with an important modification. While in a standard CVAE different conditions are represented by means of fixed OHE vectors ${{\bf{c}}}_{i}\in {{\mathbb{Z}}}^{N}$ that are concatenated to the input ${{\bf{x}}}_{i}\in {{\mathbb{R}}}^{G}$, scPoli uses learnable embeddings ${{\bf{s}}}_{i}\in {{\mathbb{R}}}^{E}$ of fixed dimensionality *E* to represent conditions. The learning objective for this network is akin to that of a standard CVAE, but the embeddings **s** are optimized during training as parameters of the model using backpropagation:

5 $${ {\mathcal L} }_{{\mathrm{CVAE}}}(\theta ,\phi ,{\bf{s}})=-{{\mathbb{E}}}_{{\bf{z}}\sim {q}_{\phi ,{\bf{s}}}({\bf{z}}|{\bf{x}},{\bf{c}})}\log {p}_{\theta ,{\bf{s}}}({\bf{x}}|{\bf{z}},{\bf{c}})+{D}_{{\mathrm{KL}}}({q}_{\phi ,{\bf{s}}}({\bf{z}}|{\bf{x}},{\bf{c}})||p({\bf{z}}))\left).\right.$$

Condition embeddings are implemented using the torch.nn.Embedding class, which takes as input an index indicating the condition and outputs the learned embedding. These embeddings are randomly initialized and optimized together with the rest of the trainable parameters of the network by minimizing the loss function used to train the model.

### Prototypes for label transfer

scPoli posits that cell identities can be represented using prototypes. For each labeled cell type in the data, a prototype ${{\bf{p}}}_{k}\in {{\mathbb{R}}}^{D}$ is initialized, where *D* is the dimensionality of the latent space of the CVAE model and *k* represents the cell type label. Prototypes are computed by averaging the latent representation of the data points belonging to each particular cell type:

6 $$\begin{array}{c}\begin{array}{r}{{\bf{p}}}_{k}=\frac{1}{|{\mathscr{K}}(k)|}\mathop{\sum }\limits_{i\in {\mathscr{K}}(k)}{{\bf{z}}}_{i},\end{array}\end{array}$$

where **z**_*i*_ is the latent representation of cell *i*, *k* is the cell type label and ${\mathscr{K}}\left(k\right)$ is the set of indices of cells belonging to cell type *k*:

7 $${\mathscr{K}}\left(k\right)=\left\{i,|,{a}_{i}=k\right\}.$$

Additionally, when the model is trained on partially labeled data, a set of unlabeled prototypes are initialized after clustering the unlabeled data using the Louvain algorithm implementation in Scanpy. Assuming that each unlabeled cell is now assigned a cluster *j*, unlabeled prototypes are computed using:

8 $$\begin{array}{c}\begin{array}{r}{{\bf{p}}}_{j}^{({\mathrm{unlabeled}})}=\frac{1}{|{\mathscr{J}}(j)|}\mathop{\sum }\limits_{i\in {\mathscr{J}}(j)}{{\bf{z}}}_{i},\end{array}\end{array}$$

where **z**_*i*_ is the latent representation of cell *i*, *j* is the unlabeled cluster label and ${\mathscr{J}}\left(j\right)$ is the set of indices of cells belonging to cluster *j*:

9 $$\begin{array}{c}{\mathscr{J}}\left(j\right)=\left\{i,|,{a}_{i}=j\right\}.\end{array}$$

Unlabeled prototypes offer good reference points for downstream analyses and novel cell type annotation but are not used for the prototype loss computation.

### Prototype loss

scPoli’s training objective includes a supervised term we call prototype loss. This term has the objective of pulling together cells belonging to the same cell type towards their correspondent prototype in latent space.

10 $$\begin{array}{c}{ {\mathcal L} }_{{\mathrm{prototype}}}=\frac{1}{N}\mathop{\sum }\limits_{k=1}^{K}\mathop{\sum }\limits_{i=1}^{N}{1}_{{\mathscr{K}}(k)}({a}_{i})\,d({{\bf{z}}}_{i},{{\bf{p}}}_{k}),\end{array}$$

11 $$\begin{array}{c}{1}_{{\mathscr{K}}(k)}({a}_{i}):=\left\{\begin{array}{cc}1 & {\rm{if}}\,{a}_{i}\in {\mathscr{K}}(k)\\ 0 & {\rm{if}}\,{a}_{i}\notin {\mathscr{K}}(k)\end{array},\right.\end{array}$$

where *K* is the number of cell types in the data, *a*_*i*_ is the cell type annotation of cell *i* and $d\left(\cdot ,\cdot \right)$ is a distance function. The distance is computed only between the latent representation of a cell and the prototype of the cell type to which said cell belongs. In this work we formulate this distance as the Minkowski distance between a cell and its prototype. This distance is equivalent to the Euclidean distance when *p* = 2 and to the Manhattan distance when *p* = 1. Nonetheless, any distance metric could potentially be used.

12 $$d\left({{\bf{z}}}_{i},{{\bf{p}}}_{k}\right)={\left({\left|{{\bf{z}}}_{i}-{{\bf{p}}}_{k}\right|}^{p}\right)}^{\frac{1}{p}},$$

scPoli can be trained on multiple sets of prototypes (for example, from multiple cell type annotations) in parallel, in this case (10) is expanded as:

13 $${{\mathscr{L}}}_{{{\mathrm{prototype}}}}=\frac{1}{N}\mathop{\sum }\limits_{l=1}^{L}\mathop{\sum }\limits_{k=1}^{K}\mathop{\sum }\limits_{i=1}^{N}{{\bf{1}}}_{{\mathscr{K}}\left({k}^{\left(l\right)}\right)}\left({a}_{i}^{\left(l\right)}\right)\hspace{0.33em}d\left({{\bf{z}}}_{i},{{\bf{p}}}_{{k}^{\left(l\right)}}\right),$$

where *L* is the number of cell type annotations provided to the model and ${a}_{i}^{\left(l\right)}$ is the cell annotation index for cell *i* and annotation *l*.

### scPoli training

We describe the procedure used to train an scPoli model when building a reference of performing query-to-reference mapping below:

- Reference building
  - Pretraining: initialize *N* condition embeddings, where *N* is the number of samples present in the reference. The model receives gene expression ${{\bf{x}}}_{i}^{\left({{\mathrm{ref}}}\right)}\in {{\mathbb{R}}}^{G}$ (where *G* is the number of input features), which is concatenated to the sample embedding of the corresponding sample ${{\bf{s}}}_{i}\in {{\mathbb{R}}}^{E}$ as input (where *E* is the embedding dimensionality). When multiple condition labels are passed to the model, the embeddings for each condition $\{{{\bf{s}}}_{i}^{\left(i\right)},\ldots ,{{\bf{s}}}_{i}^{\left(k\right)}\}$ are concatenated to the input, where *k* is the number of condition covariates passed. The objective function ${{\mathscr{L}}}_{{{\mathrm{CVAE}}}}$ (5) is optimized on the reference dataset.
  - Fine-tuning: initialize cell type prototypes using (6) and optimize ${{\mathscr{L}}}_{{{\mathrm{CVAE}}}}+\eta {{\mathscr{L}}}_{{{\mathrm{prototype}}}}$, where *η* is a hyperparameter that tunes the strength of the prototype loss; store learned prototypes with the model.
- Reference mapping
  - Pretraining: freeze the weights of the encoder and decoder networks from the reference model, and initialize *M* additional sample embeddings where *M* is the number of samples in the query. These embeddings constitute the only trainable parameters when training on query data. The model receives gene expression ${{\bf{x}}}_{i}^{({\mathrm{query}})}\in {{\mathbb{R}}}^{G}$ concatenated to sample embeddings ${{\bf{s}}}_{i}\in {{\mathbb{R}}}^{E}$ as input. The model needs to be provided with the same number of condition covariates as the reference model. The objective function ${ {\mathcal L} }_{{\mathrm{CVAE}}}$ (5) is optimized on the query dataset.
  - Fine-tuning: initialize labeled prototypes in the query dataset if any labeled cells are present, the unlabeled data are clustered using the Louvain algorithm and unlabeled prototypes are initialized for the detected clusters using (8). Optimize ${ {\mathcal L} }_{{\mathrm{CVAE}}}+\eta { {\mathcal L} }_{{\mathrm{prototype}}}$; if all cells in the query are unlabeled, this learning objective is reduced to ${ {\mathcal L} }_{{\mathrm{CVAE}}}$. Unlabeled prototypes are not used for prototype loss computation and are only used for downstream analyses.

scPoli is optimized using Adam and a default learning rate of 0.001. The ratio of pretraining/fine-tuning epochs we use is 0.9, but the optimal value might vary depending on the input dataset.

### Cell type label transfer and uncertainty quantification

scPoli assigns to unlabeled cell *i* with latent representation *z*_*i*_ the cell type identity *k* of the closest prototype *p* in latent space:

14 $${a}_{i}^{({\mathrm{pred}})}=\mathop{\mathrm{arg}\;\mathrm{min}}\limits_{k\in {\mathfrak{K}}}d({{\bf{z}}}_{i},{{\bf{p}}}_{k}).$$

The minimum distance between the latent representation and any reference prototype is used as a proxy for uncertainty for unknown cell type detection.

15 $${u}_{i}=\mathop{\mathrm{min}}\limits_{k\in {\mathfrak{K}}}d({{\bf{z}}}_{i},{{\bf{p}}}_{k}),$$

where ${\mathfrak{K}}$ is the set of cell type identities present in the data.

We do not fix a default value for this uncertainty above which a cell should be classified as unknown. In our experiments, we observed the distributions of uncertainties and picked a quantile as the cutoff value. When we expected more unknown cells in the unlabeled data, for example in the cancer query, we picked lower quantiles. Another possible approach could be the one proposed by ref. ^4^. The authors held out a few labeled datasets as query and then after mapping them they looked for the most optimal uncertainty threshold by generating a receiver operating characteristic (ROC) curve tracking correct label transfer.

This uncertainty does not have an upper bound, but we offer the option to scale and normalize it to have values between 0 and 1.

### Scalability analysis

The introduction of learnable condition embeddings that replace OHE vectors to represent conditions leads to a difference in the number of trainable parameters between the scPoli CVAE and a standard CVAE network. Let *G* be the dimensionality of the gene expression input, *E* the dimensionality of condition embeddings in scPoli, *N* the number of conditions, *D* the latent space dimensionality, and *H*_enc_ and *H*_dec_ the widths of the input layers of the encoder and decoder, respectively. When comparing these two models with the same number and width of hidden layers and latent dimensionality and ignoring bias terms of the fully connected layers, scPoli introduces *E* × *N* parameters in the embedding matrix, $\left(G+E\right)\times {H}_{{{\mathrm{enc}}}}$ parameters at the input layer of the encoder and $\left(D+E\right)\times {H}_{{{\mathrm{dec}}}}$ at the decoder. A standard CVAE has $\left(G+N\right)\times {H}_{{{\mathrm{enc}}}}$ and $\left(D+N\right)\times {H}_{{{\mathrm{dec}}}}$ at the encoder and decoder input, respectively. From this, it can be derived that scPoli will have fewer trainable parameters than a standard CVAE when:

$$\begin{array}{c}E < \frac{N\times H}{N+H}\,\text{where}\,H={H}_{{{\mathrm{enc}}}}+{H}_{{{\mathrm{dec}}}}.\end{array}$$

This inequation results in

$$\begin{array}{rr}E < H & {\rm{if}}\,N\gg H\\ E < \frac{H}{2} & {\rm{if}}\,N\approx H\\ E < N & {\rm{if}}\,N\ll H.\end{array}$$

Considering the common choice of 100 or lower for H, and the common choice of values below 25 for *E*, we can see that scPoli results in a lower number of parameters in the case of a relatively high number of conditions *N* (Supplementary Fig. 14). While it will be comparable with a standard CVAE in the case of few conditions to integrate.

### Hyperparameters and training

We performed a hyperparameter search on the pancreas dataset of the benchmark datasets. We included parameters such as the depth of encoder and decoder, the weight *η* for the prototype loss, the embedding dimensionality, the latent dimensionality and the KL annealing parameter. We fixed the width of the hidden layer to be the square root of the number of features in the input data, as is done in^15^. We tried to fix as many hyperparameters as possible to keep the computational overhead within a reasonable limit. We selected the set of hyperparameters that yielded the best integration performance and then used these to obtain results for the benchmarks displayed in Fig. 2. A table with the grid of values considered during our hyperparameter search is available at Supplementary Table 1.

### Hyperparameters of trained models

A table with the hyperparameters used for training the models presented in this work is available at Supplementary Table 2.

### Computational cost analysis

We ran scPoli for 100 epochs (80 pretraining) on the same dataset (PBMC benchmarking data, 4,000 highly variable genes (HVG)) under- or oversampled to reach sizes of 1K, 10K, 100K and 1M cells. The model was trained with either a sparse or a dense input matrix to test the difference in memory consumption and training time between the two. We tracked the time needed to train the model for 100 epochs and max memory consumption during training net of the memory needed to store the data. The experiment was run on a server with two Intel Xeon Platinum 8280L 2.70 GHz central processing units and an NVIDIA v100 graphics processing unit. The results of this analysis are available in Supplementary Fig. 15.

### scATAC-seq integration

We adapted scPoli to work on scATAC-seq data by using a Poisson likelihood for modeling fragment counts. Fragment counts are obtained by aggregating odd and neighboring even reads. This approach was proposed by ref. ^37^. We ran PeakVI using comparable parameters to obtain a comparison.

### Association with PCs

We compute the association between a covariate and a PC by fitting a linear model with the covariate as the predictor and the values of the PC as dependent variable. We fit this model by using the lm() function in R. After fitting the model, we report the adjusted *R*^2^, which is defined as:

$${R}_{{{\mathrm{adj}}}}^{2}=1-(1-{R}^{2})\frac{n-1}{n-p-1},$$

where *n* is the number of samples and *p* is the number of predictors.

### Benchmarks

#### Integration methods

We benchmarked the data integration performance of our model against various state-of-the-art methods. These include:

- scArches scVI (v0.5.3): we ran the model using default parameters;
- scArches scANVI (v0.5.3): we ran the model using default parameters;
- Seurat v3 (v4.0.3): we followed the tutorial^43^ and used supervised PCA for reducing the data dimensionality to 50;
- Symphony (v0.1.0): we followed the vignette^44^ and ran the model using default parameters.

#### Cell type classification methods

We benchmarked the performance on label transfer and cell type classification against the following methods:

- scArches scANVI (v0.5.3): we ran the model using default parameters;
- MARS: we ran the model using default parameters;
- Seurat v3 (v4.0.3): we followed the vignette^43^ and ran the model using default parameters;
- SVM: we fit a LinearSVC object from scikit-learn (v0.24.2) on the reference data.

### Metrics

We quantified the quality of the data integration using the following metrics from the scIB (v 1.0.0) package and Luecken et al. Cell type ASW (average silhouette width), isolated label F1, isolated label silhouette, NMI (normalized mutual information) and ARI (adjusted Rand index) were used as biological conservation metrics. To quantify batch mixing we used PC regression, graph connectivity and batch ASW. The overall integration score is a weighted average of the average batch mixing score and the average biological conservation score, with weight 0.4 and 0.6 respectively. Descriptions for these metrics can be found in ref. ^6^. To quantify label transfer accuracy we used the weighted averaged and macro-averaged F1 score.

Weighted F1 score: average of the F1 scores obtained for each class, weighted by their support.

Macro F1 score: average of the F1 scores obtained for each class, without any weighting.

An overview of the used metrics can be found in Supplementary Table 3.

### Datasets

#### The HLCA

We obtained the HLCA core dataset from the authors of the study^4^. The dataset consists of 584,884 lung cells from 166 samples and 107 subjects. Gene expression is subset to 2,000 selected genes, which we used for model training. Different levels of cell type annotation and sample and patient metadata are curated and available.

#### PBMC atlas

The atlas contains 7,800,850 PBMC cells from 2,375 samples, representing cells from 25 datasets, 1,977 healthy or diseased donors. For model training and analysis, 10,000 HVGs were selected. A coarse annotation consisting of 14 cell types was used for initializing scPoli’s prototypes. The data and metadata were curated by scientists at the Chan-Zuckerberg Initiative and collected from refs. ^19,35,43,45–61^.

#### Schulte-Schrepping et al. dataset

This is a published^35^ PBMC dataset of 65 patients with COVID-19 and healthy controls. The dataset contains 99 samples and 222,003 cells, and was downloaded as part of the Fredhutch COVID-19 collection available at ref. ^62^. For model training and analysis 4,000 HVGs were used.

#### Su et al. dataset

This is a published^34^ PBMC dataset of 129 COVID-19 patients and 16 healthy controls. The dataset contains 270 samples and 559,517 cells, and was downloaded as part of the Fredhutch COVID-19 collection available at ref. ^62^. For model training and analysis 2,000 HVGs were used.

#### Cross-species dataset

These data consist of the transcriptomics profiling of the primary motor cortex of 305,638 single nuclei in humans, marmoset monkeys and mice^36^. The dataset was downloaded from ref. ^63^, which contains expression profiles based on the one-to-one orthologs (15,860 genes in total) defined in the three species. A total of 2,000 HVGs were selected on the basis of the reference datasets.

#### scATAC-seq dataset

We obtained the scATAC-seq data from ref. ^64^. The data were published for the NeurIPS 2021 competition on multimodal single-cell integration. We isolated only the ATAC features and trained the model on those. We filtered peaks that were detected on less than 5% of cells. After these preprocessing steps the data consisted of 69,249 cells and 16,134 features.

### Benchmark datasets

All datasets used for benchmarking were obtained from ref. ^65^ unless specified otherwise. Count data were used for all datasets.

#### Immune

The immune PBMC dataset used for benchmarking was obtained from refs. ^6,66^. The dataset contains 32,484 cells from 4 studies and 16 cell types. The data was subset to the 4,000 most highly variable genes before further analysis.

#### Pancreas

The data contain 16,382 pancreas cells from 8 different batches. The cells are annotated and assigned to 14 cell types. A total of 4,000 HVGs were used for model training and analysis.

#### Brain

The mouse brain dataset consists of 332,129 cells and 4 batches. Ten cell types are present. The data were subset to the 4,000 most highly variable genes before further analysis.

#### Endocrine

The data consist of 25,919 cells in 4 batches and 14 cell types. A total of 4,000 highly variable genes were selected for downstream analyses.

#### Tumor

The tumor atlas was obtained from refs. ^67,68^. The dataset is a collection of 14 studies on various types of cancer. It contains 317,111 cells annotated in 25 cell types. We selected 4,000 HVGs for model training.

#### Lung

The lung data were obtained from refs. ^6,66^. These data consist of 32,472 lung cells from 3 batches and 17 cell types. A total of 4,000 HVGs were used for model training.

### Reporting summary

Further information on research design is available in the Nature Portfolio Reporting Summary linked to this article.

## Online content

Any methods, additional references, Nature Portfolio reporting summaries, source data, extended data, supplementary information, acknowledgements, peer review information; details of author contributions and competing interests; and statements of data and code availability are available at 10.1038/s41592-023-02035-2.

### Supplementary information

Supplementary Information Supplementary Figs. 1–15 and Tables 1–3.

Reporting Summary

## Data Availability

All datasets analyzed in this manuscript are public and have been published in other papers. We have referenced them in the manuscript and, when necessary, made them available at http://github.com/theislab/scPoli_reproduce.

## Code availability

The software is available as part of https://scarches.readthedocs.io. The code used to generate the results is available at http://github.com/theislab/scPoli_reproduce.
