# Supplementary material for: Population-level integration of single-cell datasets enables multi-scale analysis across samples
Source: Nat Methods. 2023 Oct 9;20(11):1683–92. doi: 10.1038/s41592-023-02035-2 (PMC10630133; doi:10.1038/s41592-023-02035-2)
Supplement: Supplementary file 1 — Supplementary Figs. 1–15 and Tables 1–3. [file 41592_2023_2035_MOESM1_ESM.pdf]

nature methods

Article https://doi.org/10.1038/s41592-023-02035-2

Population-level integration of single-cell datasets enables multi-scale analysis across samples

In the format provided by the authors and unedited

List of Figures

Supplementary Figure 1 Datasets used for benchmarking. 2
Supplementary Figure 2 Stability of integration and classification performance across runs and dataset sizes. 2
Supplementary Figure 3 Integration and reference mapping with the Human Lung Cell Atlas. 3
Supplementary Figure 4 scPoli propagates high-resolution cell type labels to an under-annotated dataset in the HLCA. 3
Supplementary Figure 5 Reference mapping with the Human Lung Cell Atlas. 4
Supplementary Figure 6 Query-to-reference mapping of a cancer dataset onto the Human Lung Cell Atlas. 5
Supplementary Figure 7 Integration metrics by covariate chosen for integration in Schulte-Schrepping et al. dataset. 6
Supplementary Figure 8 scPoli can model and integrate multiple batch conditions. 6
Supplementary Figure 9 scPoli performs query-to-reference mapping across species. . . . 7
Supplementary Figure 10 scPoli can be applied to scATAC-seq integration workflows. . . . 7
Supplementary Figure 11 Exploration of the sample embedding space of a large-scale PBMC atlas. 8
Supplementary Figure 12 Principal component analysis of average gene expressions by sample in a large-scale PBMC atlas. 9
Supplementary Figure 13 Technical factors and gene patterns across samples in a large-scale PBMC atlas. 10
Supplementary Figure 14 Optimal choice for embedding dimensionality. 11
Supplementary Figure 15 Computational cost of scPoli. 11

List of Tables

Supplementary Table 1 Values and parameters tuned in the hyper-parameter search. . . . 12
Supplementary Table 2 Hyper-parameters of scPoli models used for the analyses presented in this work. 12
Supplementary Table 3 Metrics for integration and label transfer performance. 12

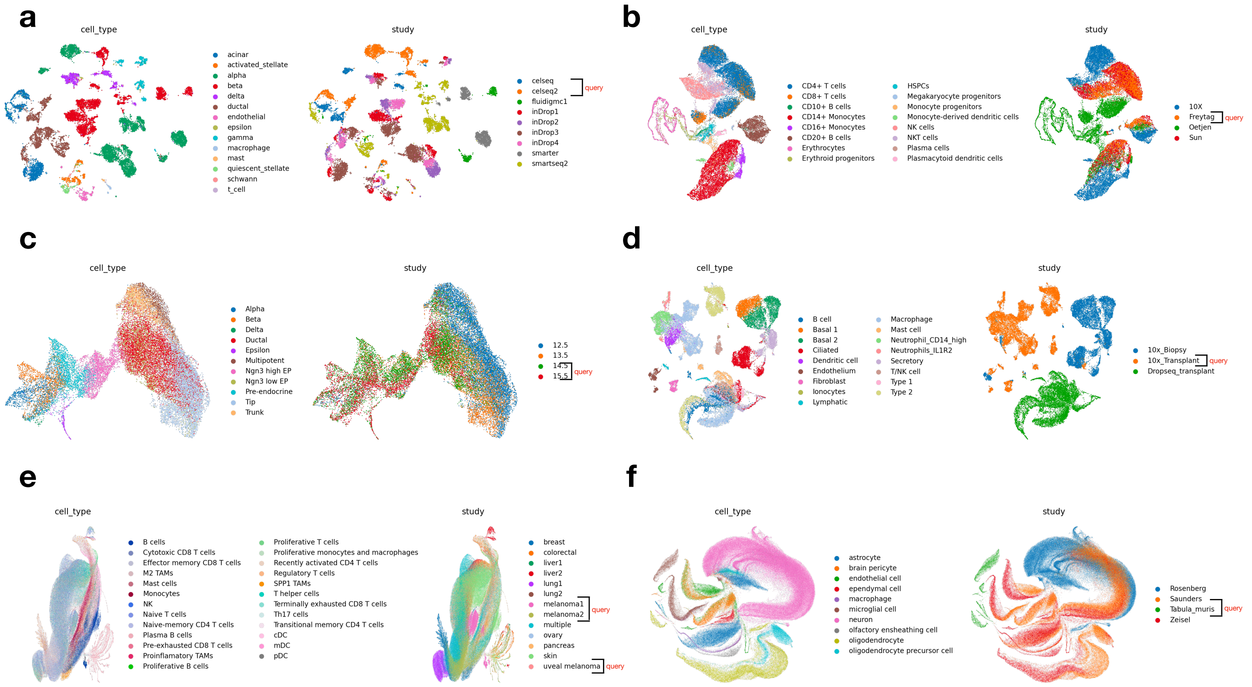

Supplementary Figure 1: **Datasets used for benchmarking. (a)** Pancreas dataset. **(b)** Immune PBMC dataset. **(c)** Endocrine dataset. **(d)** Lung dataset. **(e)** Tumor dataset. **(f)** Brain dataset.

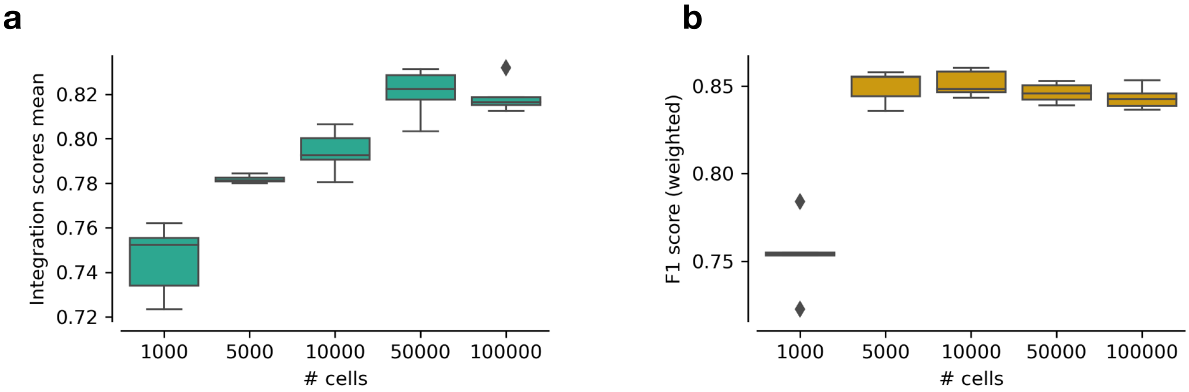

Supplementary Figure 2: **Stability of integration and classification performance across runs and dataset sizes. (a)** The results were obtained on the PBMC dataset used for benchmarking. a) Integration scores and b) classification performance obtained across reference data size. For each boxplot, there are (n=5) data points from the five runs performed at each fixed number of cells. In all boxplots, the central line denotes the median, boxes represent the interquartile range (IQR), and whiskers show the minima and maxima of the distribution excluding outliers. Outliers are all points outside 1.5 times the IQR.

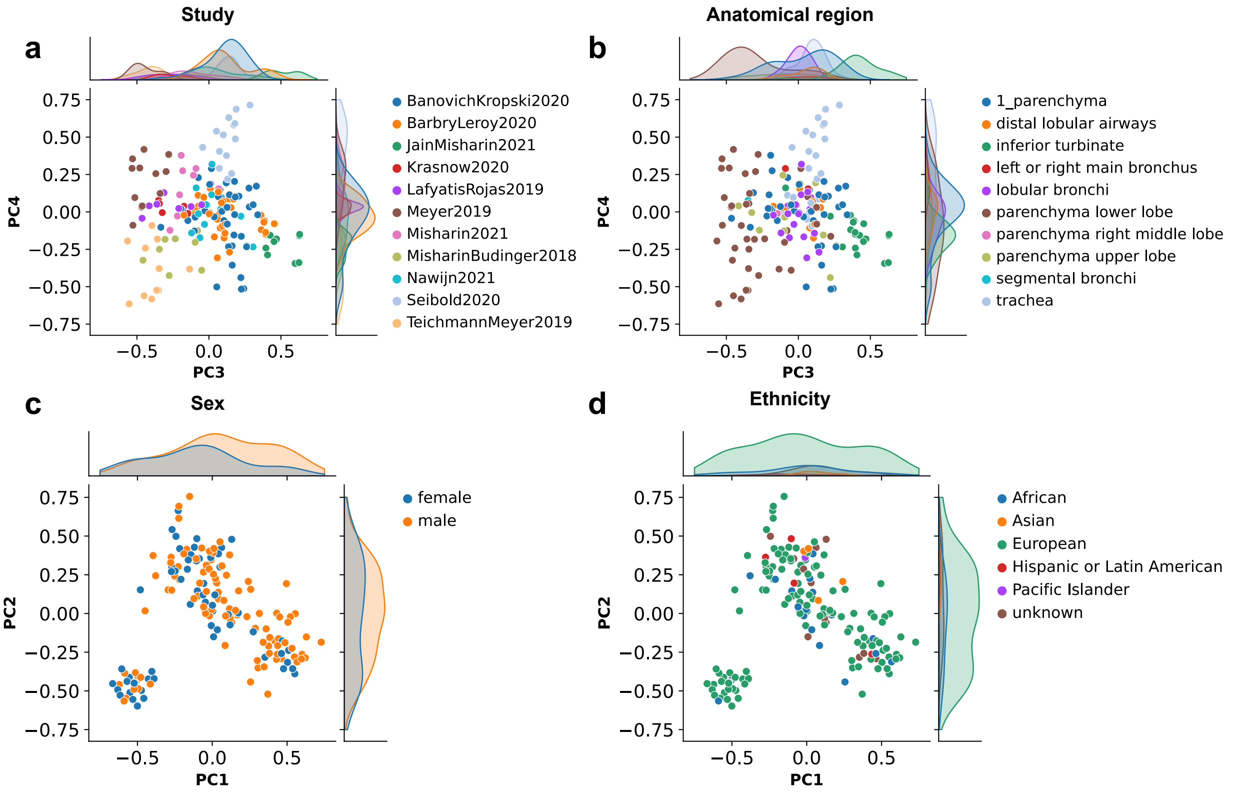

Supplementary Figure 3: **Integration and reference mapping with the Human Lung Cell Atlas.** Sample embeddings colored by study **(a)**, anatomical region **(b)**, sex **(c)**, and ethnicity **(d)**.

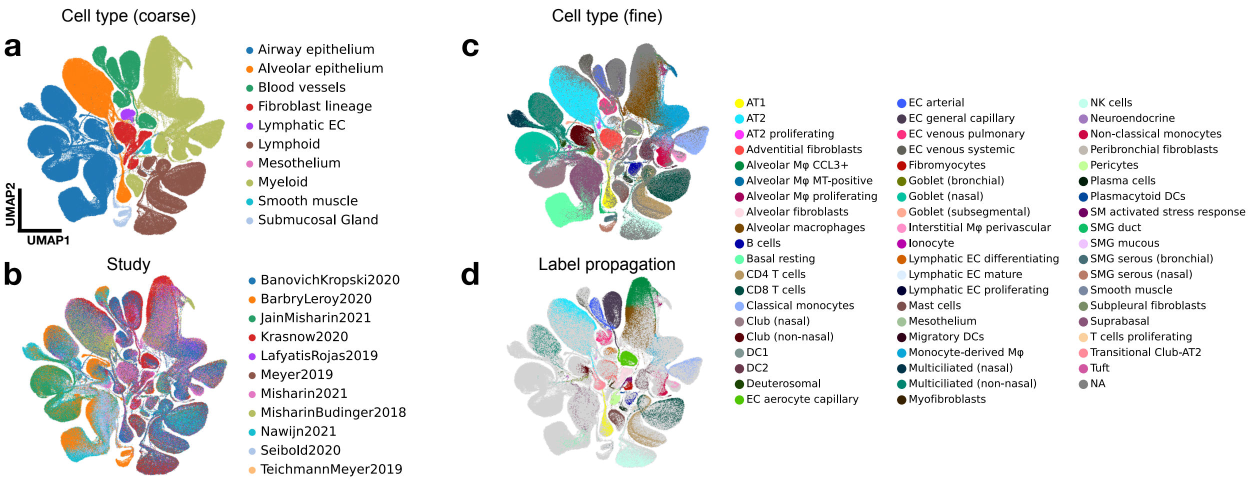

Supplementary Figure 4: **scPoli propagates high-resolution cell type labels to an under-annotated dataset in the HLCA.** **(a)** Integrated cell embeddings displaying coarse cell type annotation and **(b)** study of origin. **(c)** High-resolution annotation of the integrated studies, in grey cells missing fine annotation (Krasnow2020). **(d)** High-resolution labels are propagated to the under-annotated dataset.

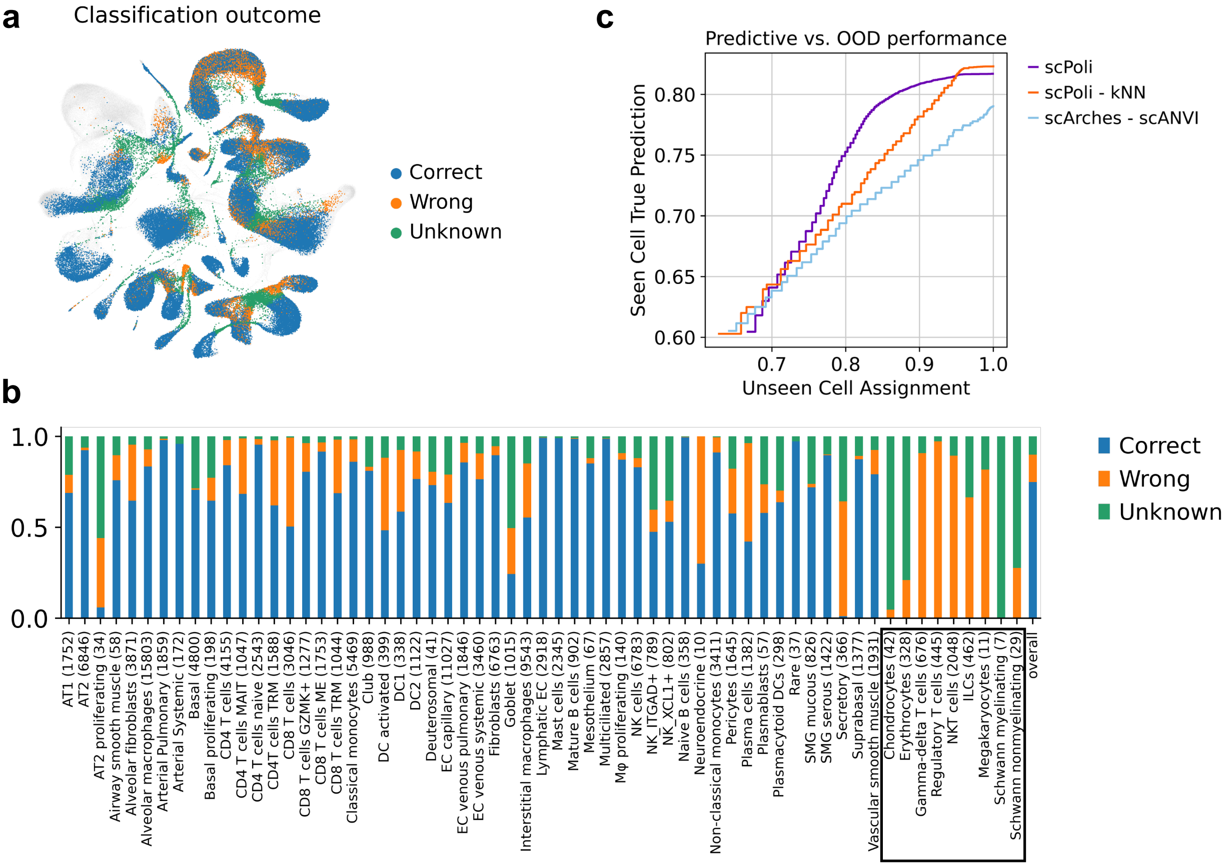

Supplementary Figure 5: **Reference mapping with the Human Lung Cell Atlas. (a)** UMAP of the joint cell embedding of reference and query with classification outcome in color. Reference cells are shown in gray. **(b)** Stacked barplot showing the accuracy of cell type classification by cell type. Cell types that were not present in the reference are marked with a box. **(c)** Label transfer performance comparison between scPoli, scPoli with a kNN classifier and scANVI. The accuracy is tracked across different uncertainty thresholds used for unknown cell type identification.

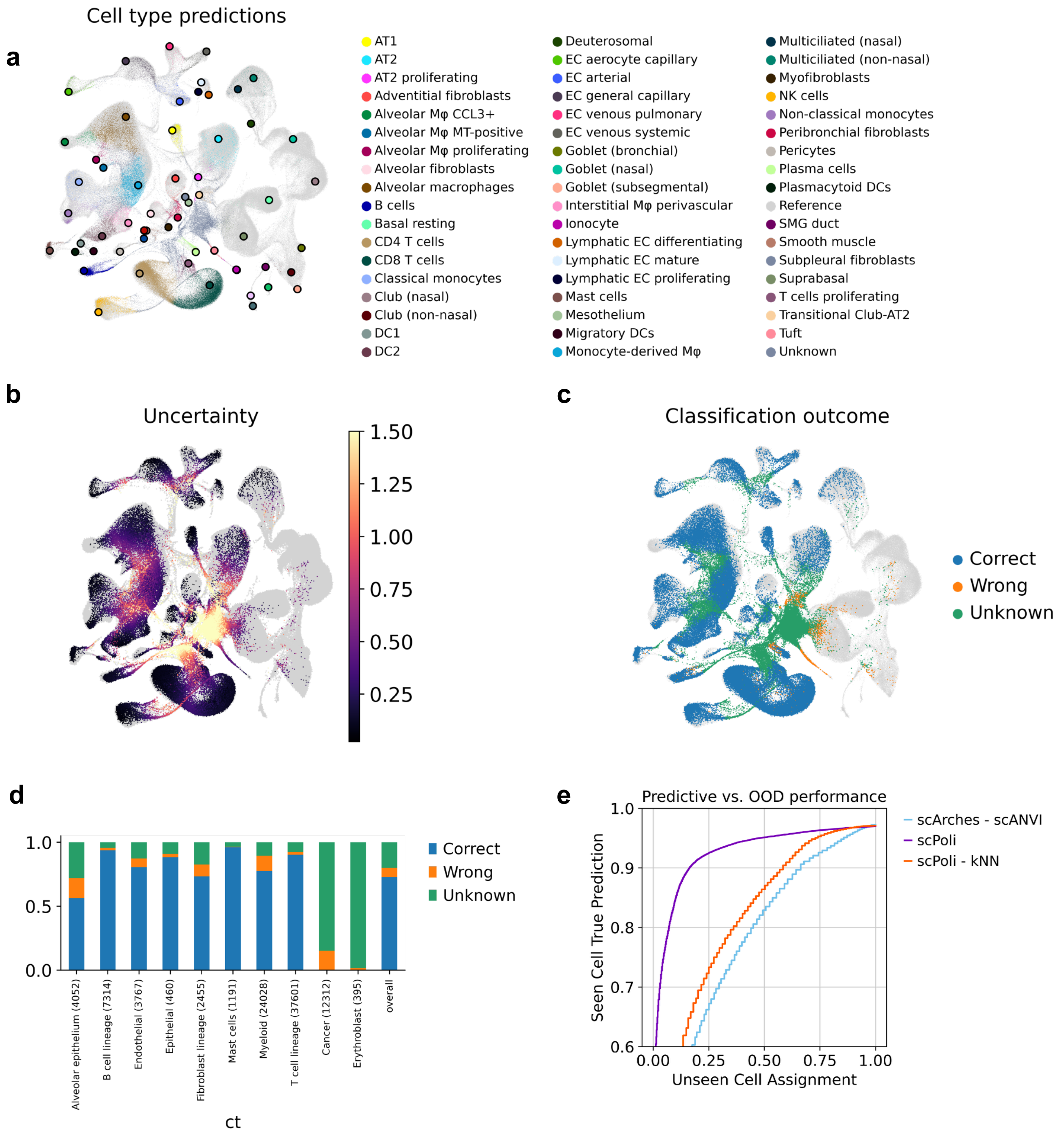

Supplementary Figure 6: **Query-to-reference mapping of a cancer dataset onto the Human Lung Cell Atlas.** **(a)** UMAP of the joint query and reference datasets after query-to-reference mapping for a cancer query. Reference cells are greyed out, while query cells are colored by the predicted cell type. Reference prototype are shown as bigger dots with a black border, and are color coded by cell type. **(b)** UMAP of the integrated object with uncertainties in color. Reference cells are shown in gray. **(c)** UMAP showing the outcome of the label transfer. Reference cells are in gray. **(d)** Stacked barplot showing the accuracy of cell type classification by cell type. Cancer cells and erythrocytes were not present in the reference data. **(e)** Label transfer performance comparison between scPoli, scPoli with a kNN classifier and scANVI. The accuracy is tracked across different uncertainty thresholds used for unknown cell type identification.

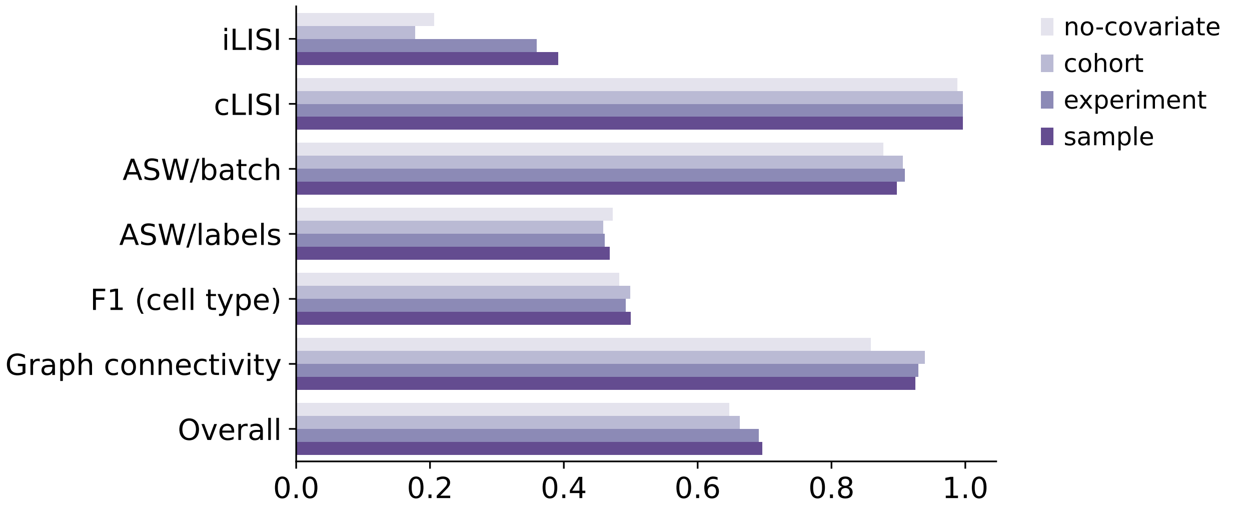

Supplementary Figure 7: **Integration metrics by covariate chosen for integration in Schulte-Schrepping et al. dataset.** Values of the integration metrics obtained after applying scPoli on the Schulte-Schrepping dataset and conditioning on different batch covariates. "No-covariate" indicates a model trained on a dummy batch covariate and relying on only prototype information for data integration.

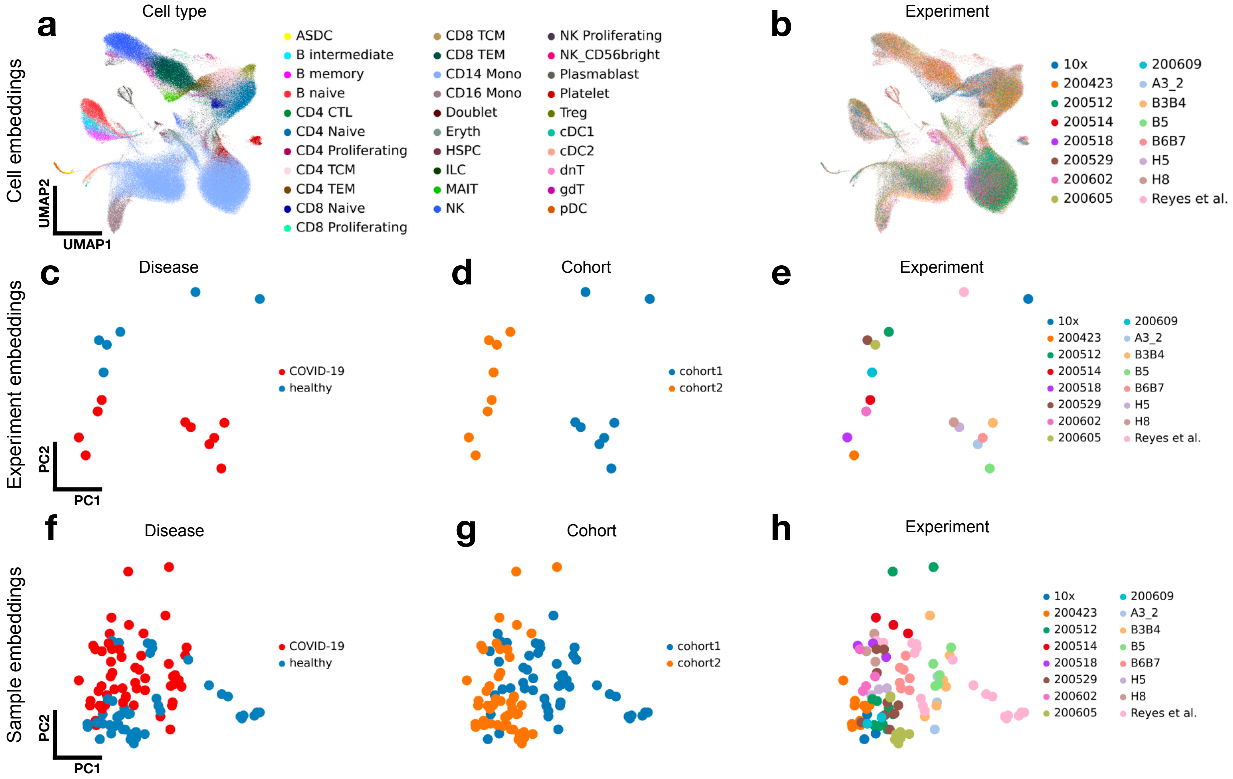

Supplementary Figure 8: **scPoli can model and integrate multiple batch conditions. (a)** Integrated cell embeddings obtained by scPoli on the Schulte-Schrepping dataset when conditioning on both "experiment" and "sample" covariates. Cells are colored by cell type label. **(b)** Integrated cell embedding, colored by experiment of origin. **(c, d, e)** Experiment embeddings colored by disease, cohort and experiment, respectively. **(f, g, h)** Sample embeddings colored by disease, cohort and experiment, respectively.

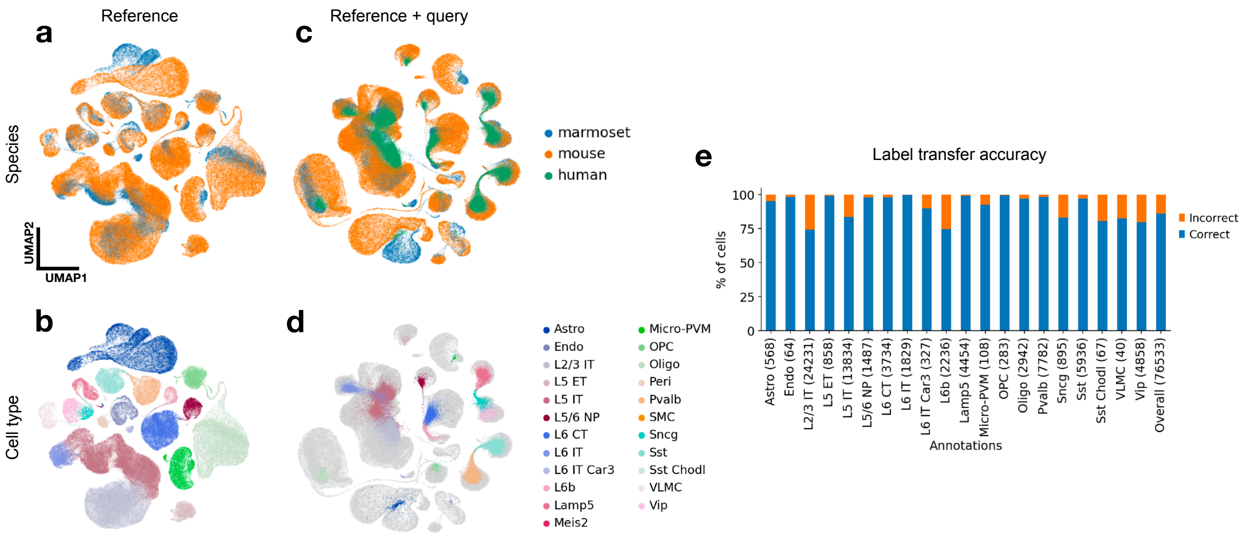

Supplementary Figure 9: **scPoli performs query-to-reference mapping across species. (a)** Integrated cell embeddings colored by species after integrating data from marmoset and mouse. **(b)** Integrated cell embedding of the built reference colored by cell type. **(c)** Integrated cell embedding after mapping a human query on top of the reference, colored by species of origin. **(d)** Label propagation from reference to the human query. Reference cells are displayed in light grey, query cells are colored by the predicted cell type label. **(e)** Accuracy of the label transfer across species by cell type.

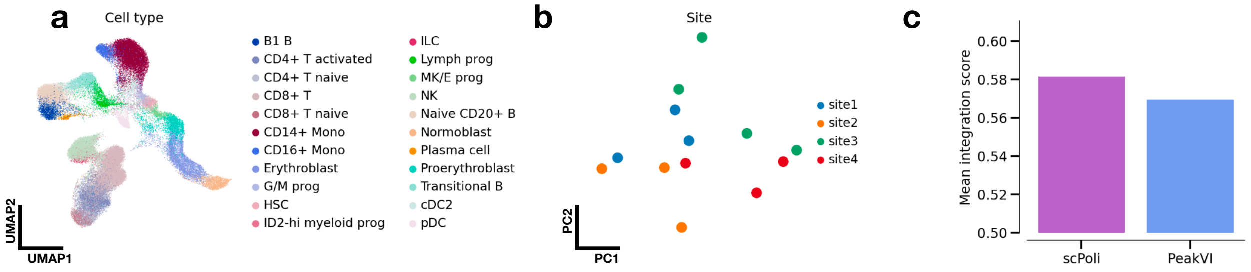

Supplementary Figure 10: **scPoli can be applied to scATAC-seq integration workflows. (a)** Integrated cell embedding yielded by scPoli on the NeurIPS 2021 competition multiome dataset (trained only on ATAC features). Cells are colored by cell types. **(b)** Sample embeddings colored by site of collection. **(c)** Integration performance comparison with PeakVI.

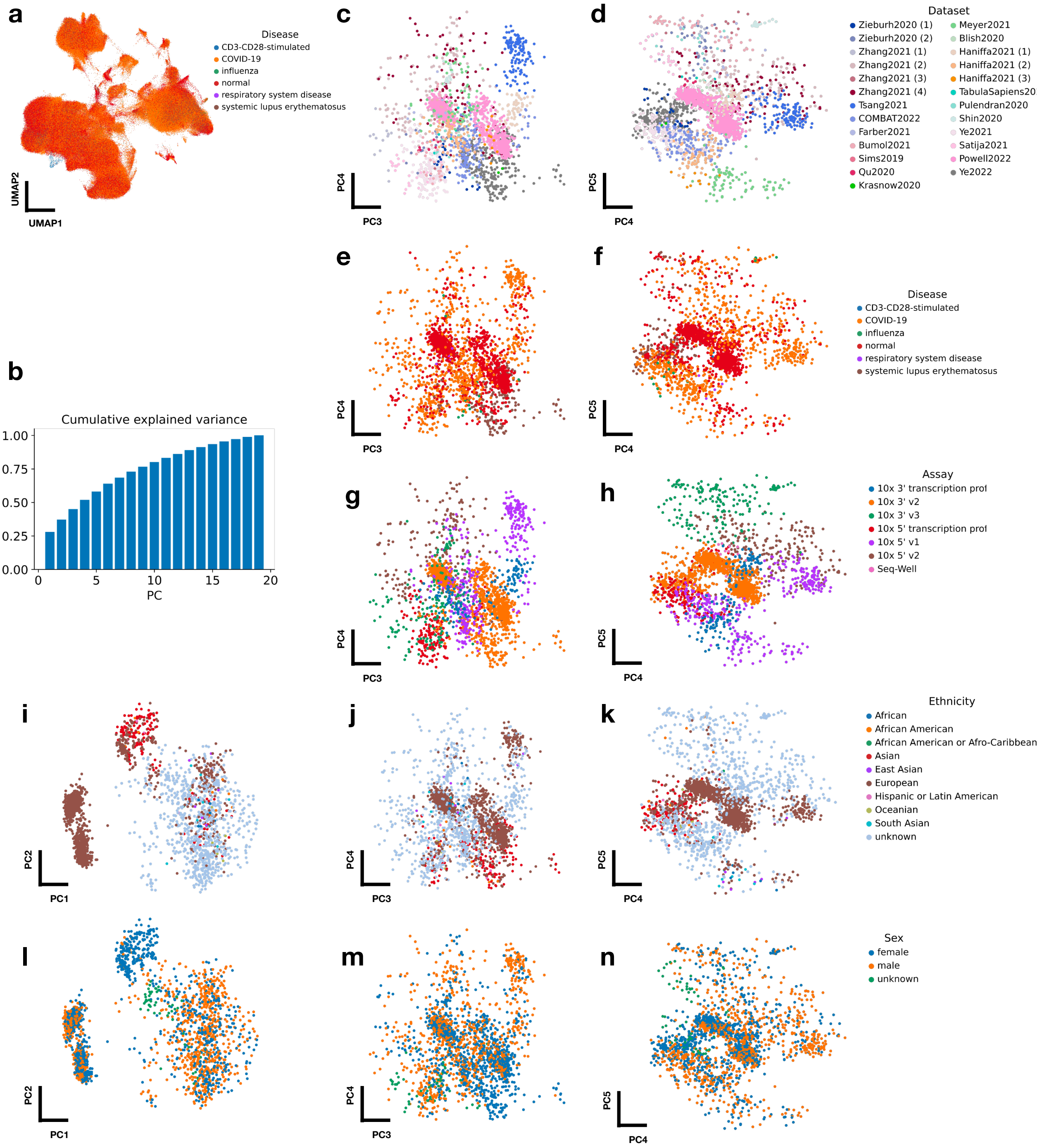

Supplementary Figure 11: **Exploration of the sample embedding space of a large-scale PBMC atlas.** **(a)** UMAP of the integrated atlas, cells are colored. by disease. **(b)** Cumulative explained variance by principal component in the sample embedding space. **(c)** Samples colored by dataset of origin in PC3 and PC4, and **(d)** PC4 and PC5. **(e)** Samples colored by disease in PC3 and PC4, and **(f)** PC4 and PC5. **(g)** Samples colored by assay in PC3 and PC4, and **(h)** PC4 and PC5. **(i, j, k, l)** Samples colored by ethnicity metadata and sex metadata **(l, m, n)** in the first 5 principal components.

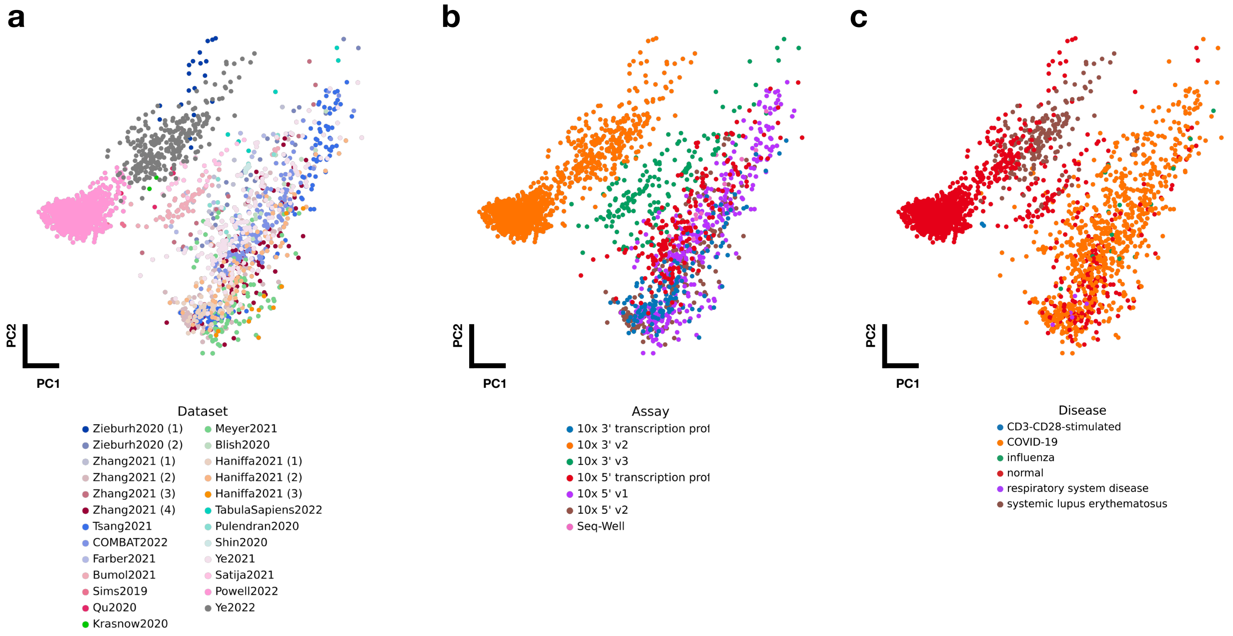

Supplementary Figure 12: **Principal component analysis of average gene expressions by sample in a large-scale PBMC atlas.** First two principal components computed on the average gene expression by sample colored by **(a)** dataset, **(b)** assay and **(c)** disease state.

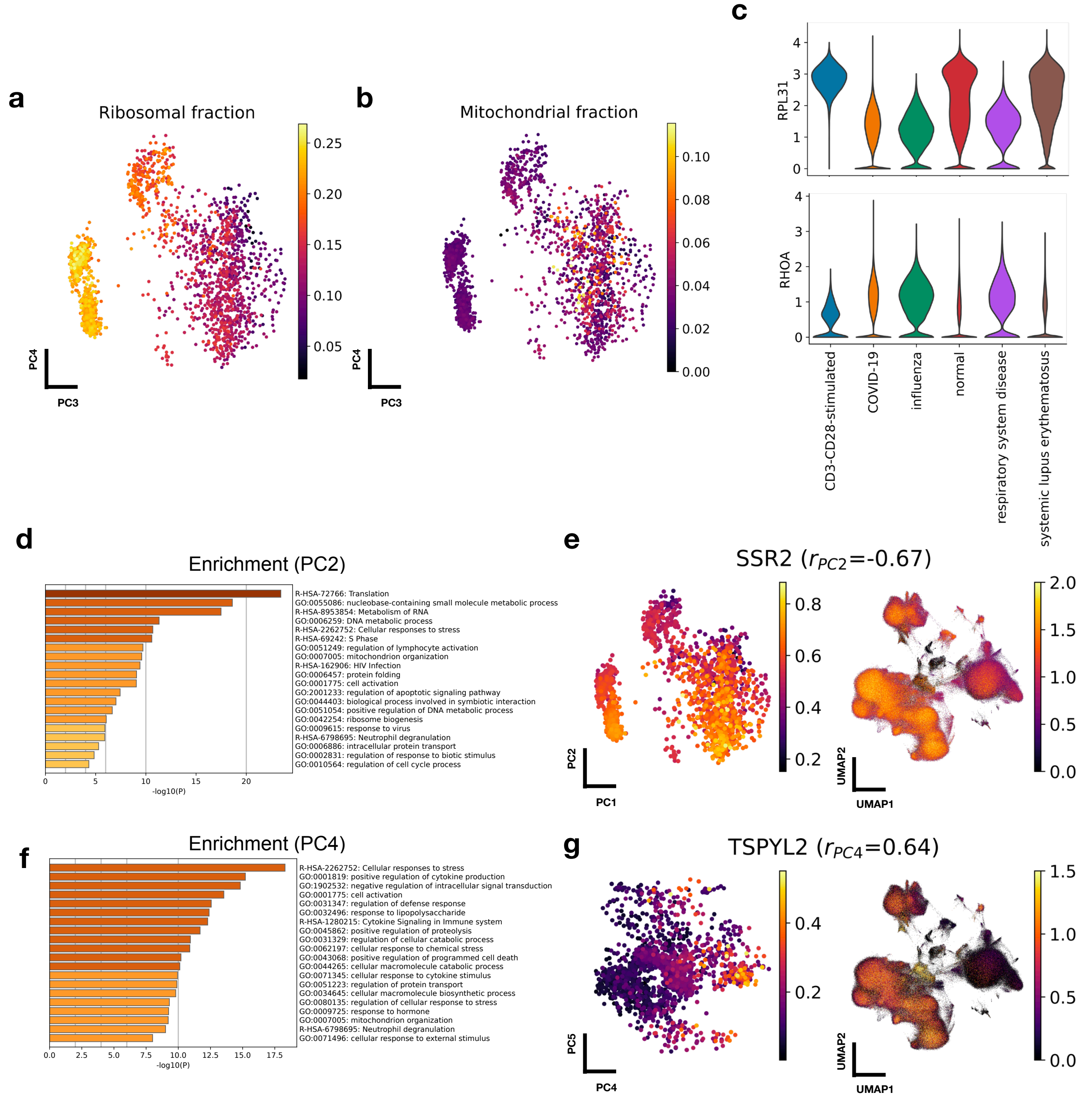

Supplementary Figure 13: **Technical factors and gene patterns across samples in a large-scale PBMC atlas.** **(a)** Mean ribosomal and **(b)** mitochondrial gene fraction in the sample embedding space. **(c)** Violin plot of expression of *RPL31* and *RHOA* by disease condition. **(d)** Biological process and pathway enrichment analysis of genes significantly correlated with PC2. A hypergeometric test with Benjamini-Hochberg correction was used. **(e)** *SSR2* expression patterns in the sample embedding space (left) and cell embedding space (right). **(f)** Biological process and pathway enrichment analysis of genes significantly correlated with PC4. A hypergeometric test with Benjamini-Hochberg correction was used. **(g)** *TSPYL2* expression patterns in the sample embedding space (left) and cell embedding space (right).

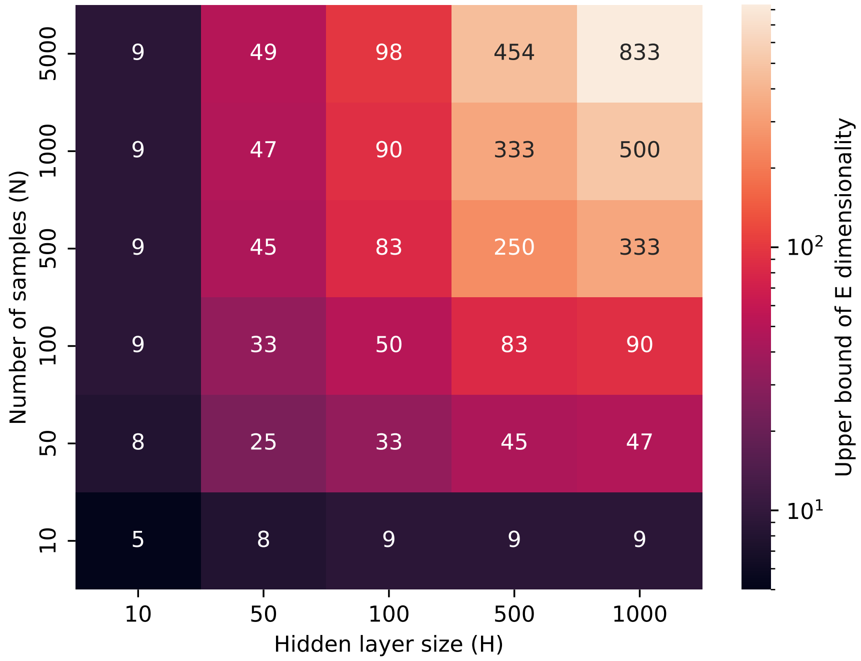

Supplementary Figure 14: **Optimal choice for embedding dimensionality.** The table displays the upper bound for the embedding dimensionality of scPoli, below which an scPoli-style CVAE scales better than a standard one. The values vary in function of the number of samples to integrate and the sum of widths of the first encoder and decoder layers.

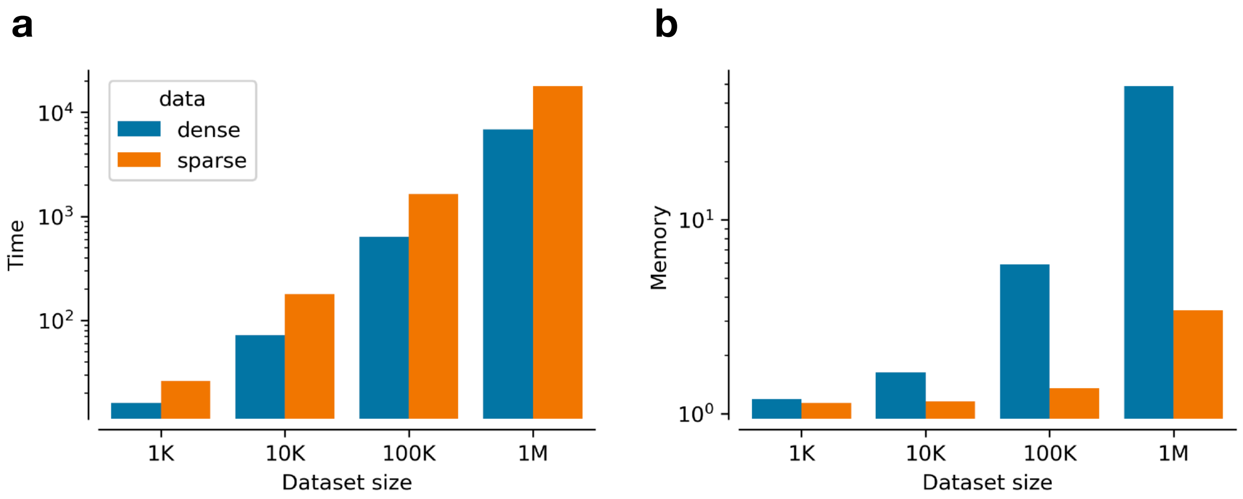

Supplementary Figure 15: **Computational cost of scPoli.** **(a)** Training time and **(b)** maximum RAM consumption of an scPoli model trained for 100 epochs (80 pre-training) on a dataset of different sizes. The model was trained on the PBMC data (4000 HVGs) used for benchmarking, sub- and super-sampled to reach the desired number of cells. The two bar hues represent values obtained when training with either dense or sparse input matrices.

Hyperparameter	Default value	Search distribution
hidden neurons, encoder and decoder	64	fixed
hidden layers	1	[1, 2, 3]
embedding dimensionality	10	[5, 10, 20]
latent dimensionality	10	[10, 25]
alpha epoch anneal	10^2	$10^{[2,3]}$
eta	1	[1, 10, 100]
p (prototype loss)	2	[1, 2]

Supplementary Table 1 | Values and parameters tuned in the hyper-parameter search.

Model	HVG	Hidden layer width	Hidden layers	Latent dim.	Embedding dim.	η	Alpha epoch anneal
Benchmarks	4000	64	1	10	5	10	100
HLCA	2000	128	3	25	20	5	100
Su *et al.*	2000	128	3	30	20	1	100
Schulte-Schrepping *et al.*	4000	128	3	30	20	1	100
PBMC atlas	10000	128	3	30	20	1	1000
ATAC	16134	100	1	25	5	0.5	100
Cross-species	2000	64	1	25	20	1	100

Supplementary Table 2 | Hyper-parameters of scPoli models used for the analyses presented in this work.

Metric	Measures
NMI	Integration (biological conservation)
ARI	Integration (biological conservation)
Cell type ASW	Integration (biological conservation)
Batch ASW	Integration (batch mixing)
Isolated label F1	Integration (biological conservation)
Isolated label silhouette	Integration (biological conservation)
Principal component regression	Integration (batch mixing)
Graph connectivity	Integration (batch mixing)
Weighted F1 score	Label transfer
Macro F1 score	Label transfer

Supplementary Table 3 | Metrics for integration and label transfer performance.
