# Supplementary material for: Population-level integration of single-cell datasets enables multi-scale analysis across samples
Source: Nat Methods. 2023 Oct 9;20(11):1683–92. doi: 10.1038/s41592-023-02035-2 (PMC10630133; doi:10.1038/s41592-023-02035-2)
Supplement: Supplementary file 2 — Reporting Summary [file 41592_2023_2035_MOESM2_ESM.pdf]

nature portfolio

Corresponding author(s): Fabian Theis

Last updated by author(s): Jul 17, 2023

Reporting Summary

Nature Portfolio wishes to improve the reproducibility of the work that we publish. This form provides structure for consistency and transparency in reporting. For further information on Nature Portfolio policies, see our Editorial Policies and the Editorial Policy Checklist.

Statistics

For all statistical analyses, confirm that the following items are present in the figure legend, table legend, main text, or Methods section.

n/a	Confirmed	
☐	☒	The exact sample size (*n*) for each experimental group/condition, given as a discrete number and unit of measurement
☒	☐	A statement on whether measurements were taken from distinct samples or whether the same sample was measured repeatedly
☐	☒	The statistical test(s) used AND whether they are one- or two-sided<br>*Only common tests should be described solely by name; describe more complex techniques in the Methods section.*
☐	☒	A description of all covariates tested
☐	☒	A description of any assumptions or corrections, such as tests of normality and adjustment for multiple comparisons
☐	☒	A full description of the statistical parameters including central tendency (e.g. means) or other basic estimates (e.g. regression coefficient) AND variation (e.g. standard deviation) or associated estimates of uncertainty (e.g. confidence intervals)
☐	☒	For null hypothesis testing, the test statistic (e.g. *F*, *t*, *r*) with confidence intervals, effect sizes, degrees of freedom and *P* value noted<br>*Give P values as exact values whenever suitable.*
☐	☒	For Bayesian analysis, information on the choice of priors and Markov chain Monte Carlo settings
☒	☐	For hierarchical and complex designs, identification of the appropriate level for tests and full reporting of outcomes
☐	☒	Estimates of effect sizes (e.g. Cohen's *d*, Pearson's *r*), indicating how they were calculated

Our web collection on statistics for biologists contains articles on many of the points above.

Software and code

Policy information about availability of computer code

Data collection	We did not use any dedicated code for data collection. All data is available, links and accession codes are provided in the data availability statement.
Data analysis	The code for scPoli is available at https://github.com/theislab/scarches. For benchmarking we used:<br>- Seurat (v4.0.2)<br>- Symphony (v0.1.0)<br>- scikit-learn (v0.24.2)<br>- scArches (v0.5.3)<br>- MARS (no version number available)<br>- scIB (v1.0.0)

For manuscripts utilizing custom algorithms or software that are central to the research but not yet described in published literature, software must be made available to editors and reviewers. We strongly encourage code deposition in a community repository (e.g. GitHub). See the Nature Portfolio guidelines for submitting code & software for further information.

Data

Policy information about availability of data

All manuscripts must include a data availability statement. This statement should provide the following information, where applicable:

- Accession codes, unique identifiers, or web links for publicly available datasets
- A description of any restrictions on data availability
- For clinical datasets or third party data, please ensure that the statement adheres to our policy

The datasets used for the study are available at:
- HLCA: https://cellxgene.cziscience.com/collections/6f6d381a-7701-4781-935c-db10d30de293
- PBMC atlas: https://figshare.com/articles/dataset/pbmc_raw_h5ad_gz/21688029
- Schulte-Schrepping et al., Su et al.: https://atlas.fredhutch.org/fredhutch/covid
- cross-species: https://zenodo.org/record/6786357
- ATAC-seq: https://www.ncbi.nlm.nih.gov/geo/query/acc.cgi?acc=GSE194122
- pancreas, brain, endocrine: https://github.com/theislab/scArches-reproducibility
- immune, lung: https://doi.org/10.6084/m9.figshare.12420968
- tumor: https://zenodo.org/record/4263972

Research involving human participants, their data, or biological material

Policy information about studies with human participants or human data. See also policy information about sex, gender (identity/presentation), and sexual orientation and race, ethnicity and racism.

Reporting on sex and gender	NA
Reporting on race, ethnicity, or other socially relevant groupings	NA
Population characteristics	NA
Recruitment	NA
Ethics oversight	NA

Note that full information on the approval of the study protocol must also be provided in the manuscript.

Field-specific reporting

Please select the one below that is the best fit for your research. If you are not sure, read the appropriate sections before making your selection.

☒ Life sciences ☐ Behavioural & social sciences ☐ Ecological, evolutionary & environmental sciences

For a reference copy of the document with all sections, see nature.com/documents/nr-reporting-summary-flat.pdf

Life sciences study design

All studies must disclose on these points even when the disclosure is negative.

Sample size	We used the sample sizes of the already available datasets we used for training the model. Information on the the size of the datasets is available in the Methods section. No statistical methods were employed to determine sample size.
Data exclusions	The single cell datasets were processed according to established best practices. This includes filtering cells by number of counts and selecting highly variable genes. These steps are mentioned in the Methods section. No samples were removed altogether.
Replication	We provide the code necessary for replicating the results. Different package versions or computational environments might lead to slightly different outputs.
Randomization	Random train/test set splits were used when training models in order to evaluate them, as common practice.
Blinding	We used public datasets to generate our results, any information regarding blinding in their study design can be found in the relevant papers. Blinding is not applicable to our work, since our main finding is the development of a computational tool.

Reporting for specific materials, systems and methods

April 2023

We require information from authors about some types of materials, experimental systems and methods used in many studies. Here, indicate whether each material, system or method listed is relevant to your study. If you are not sure if a list item applies to your research, read the appropriate section before selecting a response.

Materials & experimental systems

n/a	Involved in the study
☒	☐ Antibodies
☒	☐ Eukaryotic cell lines
☒	☐ Palaeontology and archaeology
☒	☐ Animals and other organisms
☒	☐ Clinical data
☒	☐ Dual use research of concern
☒	☐ Plants

Methods

n/a	Involved in the study
☒	☐ ChIP-seq
☒	☐ Flow cytometry
☒	☐ MRI-based neuroimaging
